# Drivers of future alien species impacts: An expert‐based assessment

**DOI:** 10.1111/gcb.15199

**Published:** 2020-07-14

**Authors:** Franz Essl, Bernd Lenzner, Sven Bacher, Sarah Bailey, Cesar Capinha, Curtis Daehler, Stefan Dullinger, Piero Genovesi, Cang Hui, Philip E. Hulme, Jonathan M. Jeschke, Stelios Katsanevakis, Ingolf Kühn, Brian Leung, Andrew Liebhold, Chunlong Liu, Hugh J. MacIsaac, Laura A. Meyerson, Martin A. Nuñez, Aníbal Pauchard, Petr Pyšek, Wolfgang Rabitsch, David M. Richardson, Helen E. Roy, Gregory M. Ruiz, James C. Russell, Nathan J. Sanders, Dov F. Sax, Riccardo Scalera, Hanno Seebens, Michael Springborn, Anna Turbelin, Mark van Kleunen, Betsy von Holle, Marten Winter, Rafael D. Zenni, Brady J. Mattsson, Nuria Roura‐Pascual

**Affiliations:** ^1^ Department of Botany and Biodiversity Research University of Vienna Vienna Austria; ^2^ Centre for Invasion Biology Department of Botany and Zoology Stellenbosch University Matieland South Africa; ^3^ Department of Biology University of Fribourg Fribourg Switzerland; ^4^ Great Lakes Laboratory for Fisheries and Aquatic Sciences Fisheries & Oceans Canada Burlington ON Canada; ^5^ Centre for Geographical Studies Institute of Geography and Spatial Planning ‐ IGOT University of Lisbon Lisbon Portugal; ^6^ School of Life Sciences University of Hawaii at Manoa Honolulu HI USA; ^7^ Institute for Environmental Protection and Research ISPRA Rome Italy; ^8^ IUCN SSC Invasive Species Specialist Group Rome Italy; ^9^ Biodiversity Informatics Group African Institute for Mathematical Sciences Cape Town South Africa; ^10^ Centre for Invasion Biology Department of Mathematical Sciences Stellenbosch University Matieland South Africa; ^11^ International Initiative for Theoretical Ecology London UK; ^12^ The Bio‐Protection Research Centre Lincoln University Christchurch New Zealand; ^13^ Leibniz‐Institute of Freshwater Ecology and Inland Fisheries (IGB) Berlin Germany; ^14^ Institute of Biology Freie Universität Berlin Berlin Germany; ^15^ Berlin‐Brandenburg Institute of Advanced Biodiversity Research (BBIB) Berlin Germany; ^16^ Department of Marine Sciences University of the Aegean Mytilene Greece; ^17^ Department of Community Ecology Helmholtz Centre for Environmental Research — UFZ Halle Germany; ^18^ Geobotany and Botanical Garden Martin Luther University Halle–Wittenberg Halle Germany; ^19^ German Centre for Integrative Biodiversity Research (iDiv) Halle – Jena –Leipzig Leipzig Germany; ^20^ Department of Biology McGill University Montreal QC Canada; ^21^ School of Environment McGill University Montreal QC Canada; ^22^ US Forest Service Northern Research Station Morgantown WV USA; ^23^ Faculty of Forestry and Wood Sciences Czech University of Life Sciences Prague Czech Republic; ^24^ CAS Key Laboratory of Marine Ecology and Environmental Sciences Institute of Oceanology Chinese Academy of Sciences Qingdao China; ^25^ Great Lakes Institute for Environmental Research University of Windsor Windsor ON Canada; ^26^ Department of Natural Resources Sciences The University of Rhode Island Kingston RI USA; ^27^ Grupo de Ecología de Invasiones INIBIOMA, CONICET – Universidad Nacional del Comahue Bariloche Argentina; ^28^ Laboratorio de Invasiones Biológicas (LIB) Facultad de Ciencias Forestales Universidad de Concepción Concepción Chile; ^29^ Institute of Ecology and Biodiversity Santiago Chile; ^30^ Institute of Botany Department of Invasion Ecology Czech Academy of Sciences Průhonice Czech Republic; ^31^ Department of Ecology Faculty of Science Charles University Prague Czech Republic; ^32^ Department of Biodiversity and Nature Conservation Environment Agency Austria Vienna Austria; ^33^ UK Centre for Ecology & Hydrology Wallingford UK; ^34^ Smithsonian Environmental Research Center Edgewater MD USA; ^35^ School of Biological Sciences University of Auckland Auckland New Zealand; ^36^ Environmental Program Rubenstein School of Environment and Natural Resources University of Vermont Burlington VT USA; ^37^ Department of Ecology and Evolutionary Biology & Institute at Brown for Environment and Society Brown University Providence RI USA; ^38^ Senckenberg Biodiversity and Climate Research Centre Frankfurt Germany; ^39^ Department of Environmental Science and Policy University of California, Davis Davis CA USA; ^40^ Ecologie Systématique Evolution AgroParisTech CNRS Université Paris‐Saclay Orsay France; ^41^ Department of Geography King’s College London London UK; ^42^ Ecology, Department of Biology University of Konstanz Konstanz Germany; ^43^ Zhejiang Provincial Key Laboratory of Plant Evolutionary Ecology and Conservation Taizhou University Taizhou China; ^44^ Division of Environmental Biology National Science Foundation Alexandria VA USA; ^45^ Department of Biology Federal University of Lavras Lavras Brazil; ^46^ Institute of Wildlife Biology and Game Management University of Natural Resources and Life Sciences Vienna Austria; ^47^ Departament de Ciències Ambientals Universitat de Girona Girona Spain

**Keywords:** biological invasions, expert survey, globalization, impacts, management, policy, scenarios, uncertainties

## Abstract

Understanding the likely future impacts of biological invasions is crucial yet highly challenging given the multiple relevant environmental, socio‐economic and societal contexts and drivers. In the absence of quantitative models, methods based on expert knowledge are the best option for assessing future invasion trajectories. Here, we present an expert assessment of the drivers of potential alien species impacts under contrasting scenarios and socioecological contexts through the mid‐21st century. Based on responses from 36 experts in biological invasions, moderate (20%–30%) increases in invasions, compared to the current conditions, are expected to cause major impacts on biodiversity in most socioecological contexts. Three main drivers of biological invasions—transport, climate change and socio‐economic change—were predicted to significantly affect future impacts of alien species on biodiversity even under a best‐case scenario. Other drivers (e.g. human demography and migration in tropical and subtropical regions) were also of high importance in specific global contexts (e.g. for individual taxonomic groups or biomes). We show that some best‐case scenarios can substantially reduce potential future impacts of biological invasions. However, rapid and comprehensive actions are necessary to use this potential and achieve the goals of the Post‐2020 Framework of the Convention on Biological Diversity.

## Graphical abstract

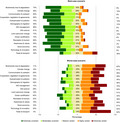

## 1 INTRODUCTION

The impacts caused by alien species on biodiversity and human livelihoods are substantial (Bacher et al., 2018; IPBES, 2019; Shackleton, Shackleton, & Kull, 2019; Simberloff et al., 2013; Vilà et al., 2011), and the numbers of alien organisms are still increasing worldwide (Seebens et al., 2017, 2018). Accordingly, much research effort has been devoted to understanding the historical trajectories of alien species accumulation, their impacts and the underlying drivers (e.g. Dawson et al., 2017; Dyer et al., 2017; Seebens et al., 2017; Vilà et al., 2011). What is lacking, however, is an assessment and understanding of the potential future impacts of alien species on biodiversity and human livelihoods (Lenzner et al., 2019; Roura‐Pascual, Richardson, Chapman, Hichert, & Krug, 2011). This is in stark contrast to other drivers of global biodiversity loss, such as climate or land‐use change, for which detailed assessments of potential future impacts have been developed (Hurtt et al., 2011; Moss et al., 2010).

This gap persists for several reasons. First, biological invasions, like other global change aspects, are a complex and context‐dependent phenomenon; so far limited data availability severely constrained the development of general predictive models, especially because of the need to consider large areas, long time periods and a large number of alien species across many taxonomic groups and habitat types. Second, impacts caused by alien species on biodiversity (Blackburn et al., 2014) and human livelihoods (Bacher et al., 2018) differ markedly among invaded regions, and variations in perceptions, values and interests provide additional context and further complicate the assessment and projection of impacts (Essl et al., 2017). This context dependency largely affects and complicates coordinated management efforts of biological invasions across regions and scales (Crowley, Hinchliffe, & MacDonald, 2017; Epanchin‐Niell et al., 2010). Finally, in most cases, there are large uncertainties about how a given alien species (or group of alien species) will respond in range and abundance to particular changes in the environment or human activities, and how such changes in distribution will affect interactions with resident biota and human activities that may ultimately translate into impacts (Hui & Richardson, 2019). Consequently, quantitative projections of how biological invasions may unfold in the decades to come under alternative trajectories of environmental change are missing (IPBES, 2016; Lenzner et al., 2019).

While the development of quantitative models to analyse the range of potential future impacts of alien species is challenging due to the complex interactions underlying biological invasions, other approaches that can shed light on future trajectories of biological invasions are more feasible. In particular, different methods, such as horizon scanning (Roy et al., 2018; Sutherland et al., 2018), the Delphi approach (MacMillan & Marshall, 2006), analytical hierarchy processes (Drescher et al., 2013) or Bayesian networks (Uusitalo, 2007), capture expert knowledge and generate predictions for potential future developments of specific components of global environmental change and have been successfully applied (e.g. Rowland, Cross, & Hartmann, 2014). Recently, expert elicitation has been used to identify future emerging issues in biological invasions (Ricciardi et al., 2017), create a watch list of future invaders (Roy et al., 2018) and identify priority issues in invasion science and management (Caffrey et al., 2014; Dehnen‐Schmutz et al., 2018).

Here, we provide an assessment of how particular drivers may affect biological invasions in contrasting contexts and under different scenarios over the next three decades (until 2050), drawing upon the knowledge of 36 biological invasions experts. Specifically, we address the following questions: (a) What is the minimum proportional increase from the current state of biological invasions that will cause major impacts on biodiversity? Furthermore, we construct two alternative futures, that is, plausible best‐case and worst‐case scenarios, both regarding the 15 most relevant drivers of future potential impacts of biological invasions in different contexts. Then, we ask (b) how likely is it that individual drivers will enable such major impacts on the environment under a best‐ or worst‐case scenario?

## 2 MATERIALS AND METHODS

Before providing a detailed description, we summarize our approach that consisted of the following four main steps. (a) We began by developing invasion scenarios under plausible futures of socio‐economic development and identifying drivers of invasions through a facilitated workshop with 25 experts. (b) Following the workshop, we developed contrasting scenarios of the drivers through the mid‐21st century. (c) We then developed and administered a survey to elicit expert judgements about thresholds for major impacts of invasions on biodiversity along with likelihoods that potential impacts of alien species will exceed these thresholds under each driver scenario. (d) Finally, we conducted statistical analyses of the survey data to examine the research questions.

### 2.1 Identification of most important drivers of biological invasions

An interdisciplinary group of 25 scholars consisting of experts of invasion science, land‐use change, global change, environmental scenario construction, elicitation processes and environmental politics convened in a workshop on invasion scenarios in Vienna, Austria, in October 2016. This workshop and subsequent work focused on laying the ground for developing invasion scenarios, that is, plausible scenarios representing how biological invasions might develop under contrasting socio‐economic and societal conditions until the mid‐21st century (Essl et al., 2019; Lenzner et al., 2019; Roura‐Pascual et al., in prep.).

An exhaustive list of putatively relevant drivers for biological invasions had been compiled in preparation for the above‐mentioned scenarios workshop. From this long list of putatively relevant drivers, the workshop participants identified and preselected a set of 15 drivers (sensu IPBES, 2016) as highly relevant for biological invasions. The 15 drivers were grouped into six broader categories: (a) global abiotic environmental change (climate change, ocean acidification, eutrophication & pollution); (b) global biotic environmental change (biodiversity loss & degradation); (c) socio‐economic activities (trade & transport, land use/cover change, socio‐economic development, demography and migration); (d) societal awareness, values, lifestyle (recreation & tourism, awareness & values, communication & outreach); (e) science, innovation and technology (invasion science, technology & innovation); and (f) societal response to invasions (cooperation, legislation & agreements, alien species management). For a more detailed description of the drivers, see Supplementary Material 1.

### 2.2 Selection of respondents and performing the survey

The first author of this study compiled a list of potential participants for the survey aiming for a balanced composition in terms of geographic regions, career stages and complementary expertise (taxonomic, geographic, environment, research focus). This resulted in a list of 50 experts of invasion science who were invited to contribute to the survey; 36 of them completed the survey between December 2017 and March 2018 (72% response rate).

The survey was circulated as an Excel workbook (Supplementary Material 2, Table A) to potential respondents. Using an offline survey was the most practical option in a pretest of the survey, allowing the respondents to revisit their assessments during any stage of completing the survey. First, respondents were asked to score the list of 15 preselected key drivers (Table 1) proposed to shape biological invasions until the mid‐21st century (2050) under contrasting socioecological contexts, and to assess the importance and uncertainty for each driver. Definitions of categories for each survey question were provided by the coordinator (F.E.) in a separate document that was circulated alongside the table (see survey instructions in Supplementary Material 1, Table B). Second, respondents were asked to provide a self‐assessment of their background and expertise (Supplementary Material 3). Overall, highest expertise among participants was concentrated in Europe (58% of the respondents) and North America (47%) followed by South America (17%), the Pacific Islands (17%), Australia (14%), Africa (14%) and Asia (11%) and taxonomic expertise was highest for plants (61%), invertebrates (47%), followed by vertebrates (44%) and microorganisms (14%). Expertise by realm was strongest in terrestrial (78%) regions followed by freshwater (36%) and marine (19%).

**TABLE 1:**
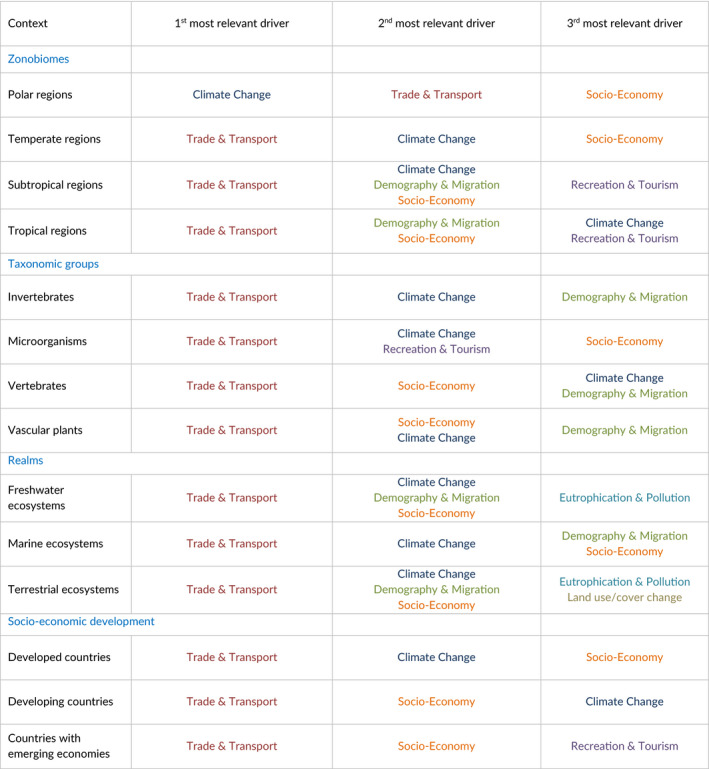
Top three most important drivers of alien species impacts until 2050 under the best‐case scenario. The ranking is context dependent and based on the coefficient estimates of the ordinal logistic regression models fit to survey data from 36 experts (see Supplementary Material 5A). Each different driver is highlighted by an individual color to increase readibility

### 2.3 Assessment of thresholds of major impacts on biodiversity

Respondents were asked to provide a threshold of the increase in invasive alien species impacts compared to current conditions that would cause a ‘major negative impact’ on biodiversity in a specific socioecological (i.e. environmental, taxonomic and socio‐economic) context by the year 2050 (see survey instructions in Supplementary Material 1). We provided them with a definition of ‘major negative impact’ on biodiversity as any ‘*substantial change in community composition*’, such as local extinction of at least one native species, severe decline of several native species, or substantial changes in ecosystem properties (structure, complexity, functioning; Blackburn et al., 2014, modified). Along with this assessment, respondents provided an uncertainty estimate on a five‐point Likert scale (1 = extremely uncertain, 2 = moderately uncertain, 3 = medium certain, 4 = highly certain, 5 = extremely certain) providing additional information on the assumed uncertainty (cf. Mastrandrea et al., 2011).

### 2.4 Developing contrasting scenarios for drivers of biological invasions

We considered a wide range of plausible changes in the impacts of biological invasions under potential future trajectories of relevant drivers. In particular, we explored two opposing storylines of how the most relevant drivers for biological invasions (outlined above) will develop in the next decades. The ‘best‐case’ and ‘worst‐case’ scenarios correspond to the best and worst plausible future development of the specific driver, as proposed in the most relevant global analysis of the respective driver (see Supplementary Material 1 for details). For the purpose of the survey, the best‐case and worst‐case scenarios of individual drivers were summarized with a specific focus on attributes deemed to be particularly relevant in a biological invasions context. In a few cases, fully developed global scenarios were not available (e.g. for ‘cooperation, legislation and agreements’ and for ‘alien species management’). In these cases, we constructed qualitative scenarios based on current evidence and available literature.

### 2.5 Assessment of driver importance

Respondents were asked to assess the importance of each driver by defining the probability (in %) that potential impacts of alien species, under a given socioecological context will by 2050 exceed the thresholds each respondent previously defined for causing major impacts on biodiversity, holding all other drivers at their current levels. This assessment was done separately for each possible combination of driver, socioecological context, and for the best‐case and worst‐case scenarios. Respondents provided their assessment by using a five‐point Likert scale approach with the following categories: 1 = extremely uncertain (0%–20% certain); 2 = moderately uncertain (21%–40% certain); 3 = medium certain (41%–60% certain); 4 = highly certain (61%–80% certain); 5 = extremely certain (81%–100% certain). Some drivers are only relevant in a subset of contexts, and in such cases (e.g. the driver ‘ocean acidification’ in terrestrial and freshwater environments), the combination was excluded from the questionnaire.

### 2.6 Analyses

First, we analysed expert predictions on potential impacts of alien species on biodiversity. For that purpose, we produced kernel density plots of the estimated threshold until the ‘major impact’ was reached for each respondent‐context combination. Subsequently, the median for each kernel density and the mean uncertainty estimate across all respondents were calculated for comparison among socioecological contexts. Kernel density calculations were made using the geom_density() function in the R‐package ‘ggplot2’. A bandwidth of two times the standard deviation was used to obtain a smooth fit. Subsequently, we calculated pairwise non‐parametric Kolmogorov–Smirnov tests between each category combination within each socioecological context (zonobiome, taxonomic group, realm, socio‐economic activities), to identify cases of significantly differing distributions.

In a second step, we assessed the driver importance within each socioecological context under best‐case and worst‐case scenarios. The aim was to identify which drivers the respondents classified as most important for enabling potential alien species impacts to exceed the previously defined threshold of major impacts. This was done through an ordinal logistic regression model (also known as ‘proportional odds model’; Guisan & Harrell, 2000) with a random intercept for respondent. Responses to all survey questions comprised the response variable, which was considered as an ordered factor. Predictor variables included a three‐way interaction between driver, socioecological context and scenario, as specified in the set of survey questions. The estimated log‐odds were subsequently transformed into probabilities representing levels of confidence that the driver would affect biological invasions to a degree that they surpass the threshold of major impacts on biodiversity. We fit this full model to all survey responses using the glmer() function in the R package ‘lme4’ (Bates, 2014).

Not all driver–system–scenario combinations were scored by respondents resulting in convergence problems in the ordinal logistic regression model. For that reason, we included a ‘dummy respondent’ that answered each driver–system–scenario combination, increasing each answer combination (driver–system–scenario) by one. This procedure has some minor implications for the results. By including one additional answer to each category, those with an initially lower number of answers are weighted slightly higher than before and vice versa. Including the ‘dummy respondent’ leads to model convergence, resulting in a more conservative estimation of the probability estimates from the regression analysis and hence more reliable estimates compared to results from models with convergence problems (Heinze & Schemper, 2002).

## 3 RESULTS

### 3.1 The threshold of major impacts on biodiversity across different contexts

**FIGURE 1:**
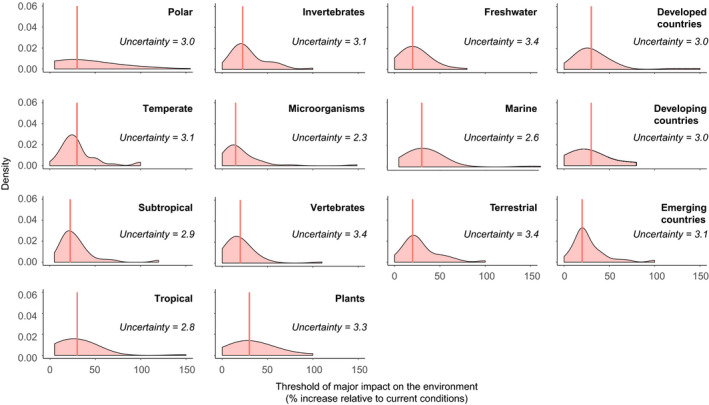
Density distribution of the increase in alien species compared to the current conditions required to cause major impacts on biodiversity, as estimated by 36 experts. Vertical red lines indicate the median value of the density distributions. Columns correspond to zonobiomes, taxonomic groups, realms and socio‐economic development (from left to right); see Supplementary Material 4. Uncertainty estimates are the mean uncertainty values provided by the experts using a five‐point Likert scale

The 36 respondents provided thresholds on what level of increase would result in future major negative impacts of alien species on biodiversity relative to the current impacts of invasive alien species for 14 different socioecological contexts (Figure 1; Supplementary Material 4). These thresholds thus provide an assessment of relative increases (in %), but not of absolute changes. Median thresholds in most contexts ranged between 20% and 30% increase compared to the current conditions (Figure 1; Supplementary Material 4). The lowest thresholds were for terrestrial and freshwater environments, countries with emerging economies and vertebrates and microorganisms (+20%), the highest were for marine environments, developed countries and countries with emerging economies, tropical, temperate and polar regions and plants (+30%). Although there are minor differences in medians among environments (i.e. freshwater, marine, terrestrial), there are moderate differences among taxonomic groups (plants have a higher median than the other taxonomic groups) and among socio‐economic contexts (countries with emerging economies having a lower median than developing and developed countries). Among climate contexts, the median is the highest for tropical climates, while polar, temperate and subtropical climates have somewhat lower medians. However, the pairwise Kolmogorov–Smirnov test showed significant differences between the density distributions of vertebrates and plants and between freshwater and marine realms. All other tests generated non‐significant results (Supplementary Material 5).

The uncertainty ratings provided by experts averaged between 2.3 (for microorganisms) and 3.4 (for vertebrates, freshwater and terrestrial environments; Figure 1). The highest uncertainties among zonobiomes were for tropical zones, microorganisms among taxonomic groups, marine among realms, whereas essentially no difference in uncertainty was observed among countries classified by socio‐economic development.

### 3.2 Driver impacts on biodiversity under best‐ and worst‐case scenarios

**FIGURE 2:**
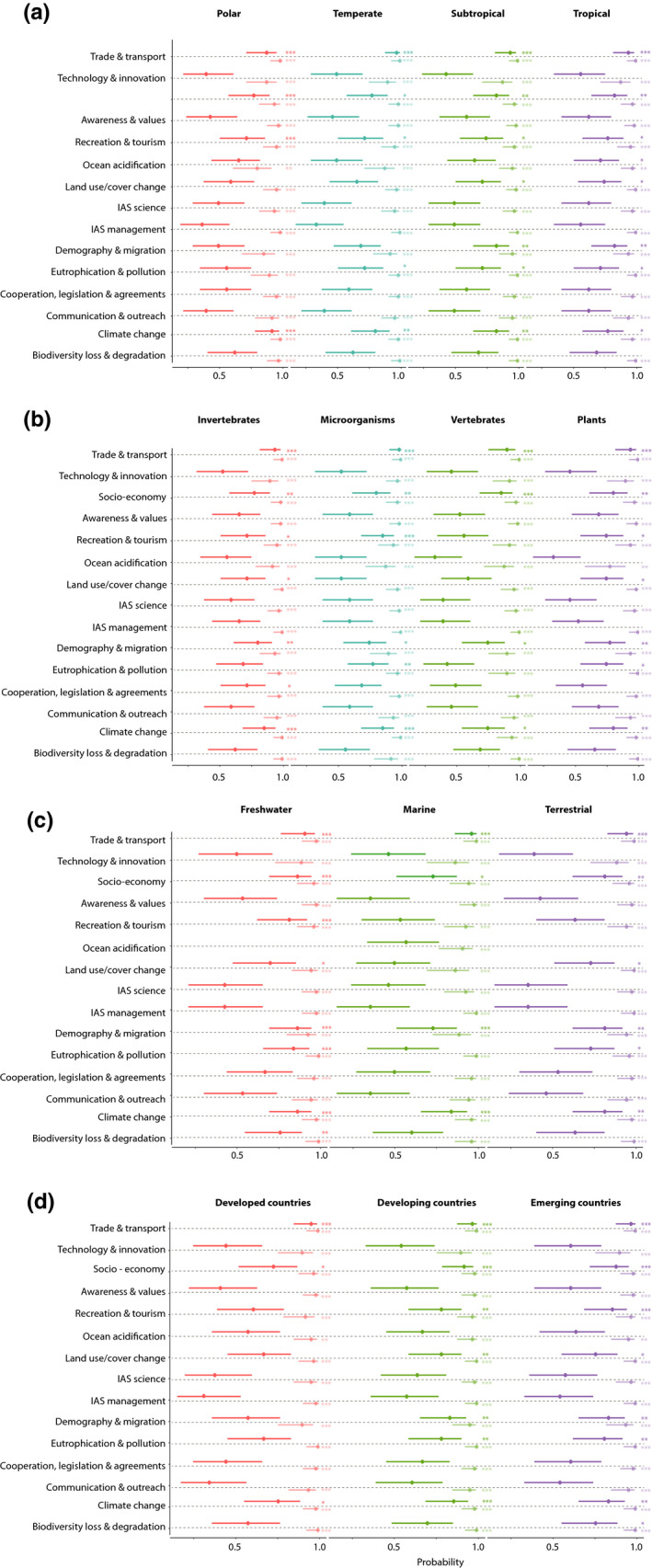
Importance of drivers of major alien species impacts on biodiversity under a best‐case and worst‐case scenario among socioecological contexts as estimated by 36 experts on biological invasions. Responses are summarized by socioecological context: (a) zonobiomes, (b) taxonomic groups, (c) realm and (d) socio‐economic development. Estimates indicate the probability of respondents answering in lower uncertainty categories, meaning they are more certain that the driver is likely to surpass the threshold of major impact on biodiversity. Significant estimates are indicated by asterisks (significance levels: * < 0.05, ** < 0.01, *** < 0.001). Darker whiskers represent estimates under a best‐case scenario for the respective drivers, and lighter whiskers represent estimates under a worst‐case scenario. In panel (d), socioecological contexts are defined as (i) developed countries: socio‐economically highly developed countries; (ii) developing countries: socio‐economically poor countries with mostly slow rates of economic growth; (iii) countries with emerging economies: socio‐economically rapidly developing countries and middle income countries (for all definitions, see Table S2)

Under the best‐case scenario for the respective drivers, trade & transport, socio‐economic development and climate change emerged as significant drivers of future biological invasions across all socioecological contexts (Table 1). Demography & migration is expected to have a significant effect in 11 socioecological contexts, that is, all except developed countries, polar regions and temperate regions. It was followed by recreation & tourism with significant effects in 10 socio‐ecological contexts (all except vertebrates, marine and terrestrial regions, and developed countries) and land use & land cover change with significant effects in eight socioecological contexts (all except polar and temperate regions, microorganisms, vertebrates, the marine realm and developed countries). Furthermore, ocean acidification emerged as a significant driver in tropical regions, while cooperation, legislation & agreements drive biological invasions by invertebrates. Finally, biodiversity loss & degradation emerged as a significant driver of biological invasions in countries with emerging economies (see Figure 2; Supplementary Materials 5 and 6).

**FIGURE 3:**
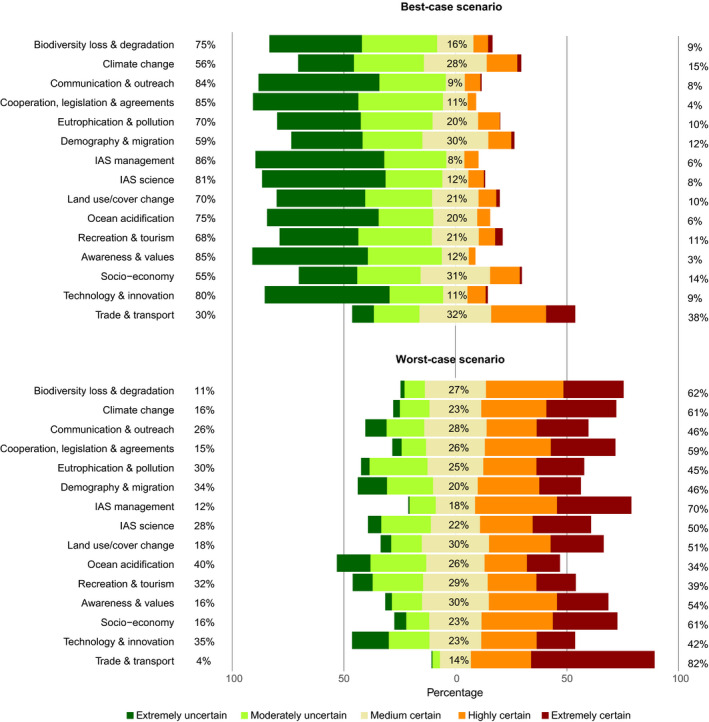
Distribution of uncertainty if 15 major drivers of biological invasions will exhibit major impacts on the environment by 2050 under a best‐ and worst‐case scenario, based on answers provided by 36 experts. The uncertainty categories follow a five‐point Likert scale. The estimates shown include all responses across 14 contexts regarding taxonomic groups, zonobiomes, realms and socio‐economic status (see Supplementary Material 1, Table 1). The stacked bars represent the uncertainty categories, with the bars and percentage value for the medium certain category centred at 0% on the *x*‐axis. Bars and percentage values on the left refer to the uncertainty categories extremely and moderately uncertain, and bars and percentage values on the right refer to the answers in the categories highly and extremely certain. Categories sum up to 100%

For the worst‐case scenarios, most respondents were certain that each driver would play a significant role in surpassing the threshold for major impact on biodiversity by alien species (Figure 3). The only driver that was not highly significant across all socioecological contexts was ocean acidification with only a medium significant effect for vascular plants, likely reflecting the paucity of species of this taxonomic group in marine environments (see Figure 2; Supplementary Material 6).

## 4 DISCUSSION

This study provides the first global assessment of potential future impacts of biological invasions on biodiversity. Specifically, we examined these potential impacts under best‐ and worst‐case scenarios in differing environmental, taxonomic and socio‐economic contexts based on a large number of drivers and considering plausible differences in how the drivers might develop (i.e. best‐ vs. worst‐case scenarios). The assessment is based on the collective knowledge across a diverse group of invasion scientists and thus reflects current understanding on the future fate of biological invasions in the Anthropocene. Experts agreed that in a worst‐case scenario, all focal drivers will contribute strongly to potential future impacts of alien species, while under the best‐case scenario, the results show a more diverse and heterogeneous pattern. Our findings therefore imply that there are substantial opportunities under best‐case scenarios to reduce potential future impacts of biological invasions. Among the three most important drivers of potential impacts of biological invasions until the mid‐21st century, respondents agreed that trade & transport, climate change and socio‐economy are consistently and highly relevant across socioecological contexts while assuming the best‐case scenario.

Trade & transport was consistently ranked as the most relevant driver in all contexts other than for polar regions (Table 1). The importance of changes in global trade for biological invasions is well known (Dawson et al., 2017; Reino et al., 2017; Sardain, Sardain, & Leung, 2019; van Kleunen et al., 2015; Winter et al., 2009). Alterations in trade (e.g. in terms of volume, regions of origin and destination, composition of traded goods) will increase the number of potential new arrivals and might increase propagule pressure (Sardain et al., 2019; Seebens et al., 2015). Changes in the global trade network may also lead to novel source pools for new alien species, and climate change will likely lead to the establishment of new trade routes (e.g. through the Arctic) that will dramatically reduce travel times and increase species survival (Eguíluz, Fernández‐Gracia, Irigoien, & Duarte, 2016; Melia, Haines, & Hawkins, 2016; Miller & Ruiz, 2014). Finally, the emergence of new trade modes (e.g. internet trade) will provide novel pathways for species trade and subsequent introduction as such pathways are likely more difficult to regulate compared to conventional modes (Humair, Humair, Kühn, & Kueffer, 2015). National and international policy on prevention efforts can be explicitly developed to counter the increased propagule pressure associated with an increase in diversity and frequency of trade routes (Reaser, Meyerson, & von Holle, 2008; Wonham, Byers, Grosholz, & Leung, 2013).

Climate change, with associated changes in mean annual temperatures, precipitation and occurrence and magnitude of extreme events, will undoubtedly shape the impacts of biological invasions on biodiversity in the future. Several modelling studies predict an increase in climatically suitable areas for alien species (e.g. Bellard et al., 2013; Dullinger et al., 2017; Gallardo & Aldridge, 2013) and increased establishment rates of alien species have been attributed to climate change, even when accounting for propagule pressure (Huang, Haack, & Zhang, 2011). However, substantial variation in the effects of climate change among geographic regions or taxonomic groups might occur. A systematic review by Bellard, Jeschke, Leroy, and Mace (2018) showed that there are also many alien plants and animals that might have less climatically suitable areas in the future. Based on the expert assessment, potential impacts from alien species invasions on biodiversity will be especially likely in polar regions. This expectation coincides with climate change projections, indicating some of the most severe effects of future climate change in these regions (IPCC, 2014).

Socio‐economic activity serves as a proxy for many human‐induced environmental changes (Essl et al., 2011; Pyšek et al., 2010). Often this variable is substituted with metrics such as per capita gross domestic product, human footprint index or human development index. These variables can be related to diverse environmental changes relevant for biological invasions, like resource and energy use, consumption or land use. With a projected future increase in global material footprint of around 75% by 2050 compared to 2015 (IRP, 2017), a substantial increase in impacts from biological invasions is very likely, as supported by the expert assessment in this study.

Aside from the three main drivers that emerged from this expert assessment, several others were deemed important in specific contexts. Human demography & migration was identified as having major impacts on biodiversity in several contexts. For tropical and subtropical regions, it was ranked as the second most important driver. In these regions, changes in human population density and migration are projected to be especially pronounced throughout the 21st century (Lutz, Butz, & Samir, 2014; Rigaud et al., 2018). Increasing human population sizes likely result in more degraded habitats and intensification of land use, which generally favour alien plant establishment and spread (Essl et al., 2011; Pyšek et al., 2010). Additionally, human intra‐ and intercontinental migration (e.g. due to climate change, economic inequalities or armed conflicts) are projected to increase (Lutz et al., 2014; Rigaud et al., 2018). Human migration has, in turn, been associated with increased spread of alien species (Di Castri, 1989).

For invertebrates, vertebrates and vascular plants, demography & migration ranked third. Invertebrates are generally spread unintentionally, in the terrestrial environment mostly as contaminants in commodities, and in the aquatic environment as stowaways in vessels (Katsanevakis et al., 2014; Pergl et al., 2017). With increasing population density and increased trade & transport, the likelihood of invertebrate introductions and subsequent spread is expected to increase (Aukema et al., 2010).

For vertebrates and vascular plants, mechanisms of invasions are more complex. While some species are introduced unintentionally as stowaways (e.g. some reptiles like the brown tree snake *Boiga irregularis* or the house gecko *Hemidactylus frenatus*, Rodda, Fritts, & Conry, 1992) or contaminants (e.g. seeds in agricultural products, Frick et al., 2011), others are introduced and subsequently spread as a result of intentional introductions from the pet (Blackburn, Dyer, Su, & Cassey, 2015; Bush, Baker, & Macdonald, 2014; Hulme et al., 2015) or horticultural (Dehnen‐Schmutz, Touza, Perrings, & Williamson, 2007; Dullinger et al., 2017; van Kleunen et al., 2018) trades. For many species, propagule pressure is much more important than their ecological characteristics (Jeschke & Starzer, 2018; Pyšek et al., 2015).

Supporting the argument that unintentional introductions increase the future risk of impacts (Pergl et al., 2017), our survey revealed that respondents consider recreation & tourism, where the argument runs along the same lines (Hulme, 2015), as an additional important driver for increased future impacts from invertebrates and microorganisms. For the latter taxonomic groups, recreation & tourism was considered as the second most important driver for potential future impacts on biodiversity. A doubling of global tourism is projected from 2010 to 2050 under the best‐case scenario (UNWTO, 2018), which will likely lead to several synergistic effects with other drivers such as infrastructure development in the respective regions (Anderson, Rocliffe, Haddaway, & Dunn, 2015). Based on our findings, recreation & tourism was an important driver in subtropical and tropical regions along with countries having emerging economies (which are mostly situated in subtropical and tropical regions). Especially in these regions, where many natural areas are still less modified by humans, increasing infrastructure development like roads—which can act as corridors for alien species—will likely lead to increased spread and potential impacts of alien species (Seebens, 2019). Furthermore, many resorts and other tourist accommodations use ornamental (often alien) plants in their green spaces. This mode of horticulture provides a significant opportunity for alien species to escape, establish and spread in the surrounding environments (Anderson et al., 2015; Pickering, Bear, & Hill, 2007).

Finally, our assessment revealed that in aquatic and terrestrial socioecological contexts, eutrophication and pollution are assumed to become a major driver of potential future impacts of alien species. Changes in ecosystem chemistry and resource availability (especially nitrogen availability) can have dramatic effects on species composition in a wide range of ecosystems (Bobbink et al., 2010). In many cases, opportunistic species, including many alien species, benefit most from higher levels of nutrient availability (Preston, Hedman, & Johnson, 2018). Results from our assessment did not indicate that eutrophication and pollution will strongly drive future invasive species impacts in marine environments. This contradicts findings from empirical investigations showing that marine litter (i.e. plastic debris) can act as a vector of alien species (Carlton et al., 2017; Rech, Borrell, & García‐Vazquez, 2016) and that marine pollution can increase invasive species success (Crooks, Chang, & Ruiz, 2011).

### 4.1 Limitations and caveats

Any expert‐based approach for identifying, circumscribing and subsequently ranking drivers of biological invasions (or, more generally, drivers affecting other complex phenomena of environmental change) is contingent on factors such as group composition, the kind of expertise, values, geographic background, gender, and interests represented in the group (Burgman, 2016; Hannagan & Larimer, 2010; Krueger, Page, Hubacek, Smith, & Hiscock, 2012; Latombe et al., 2019). This implies that expert‐based approaches cannot be fully objective, and do not necessarily represent the views of groups or individuals not involved in the survey (Nuñez et al., 2019). Nevertheless, expert‐based assessment of conservation topics has been proven to provide valuable focus for discussion and stimulate debate among the wider community (Sala et al., 2000; Sutherland et al., 2018).

In our study, we elicited the predictions of 36 experts from biological fields, with different backgrounds, expertise and interests. All respondents in the survey are leading experts in the field of invasion science. Thus, the predictions expressed represent the expertise of scientists that collectively can provide a profound understanding of the causes and consequences of biological invasions. However, still many uncertainties remain regarding how the dimensions of biological invasions may unfold in the future under contrasting scenarios for global environmental change (Lenzner et al., 2019). Predictions expressed in this survey are thus subject to personal norms, biases and uncertainties (Essl et al., 2017). Furthermore, as the group of experts is biased towards male respondents in higher academic positions with a Western (i.e. European and Northern American) background, the trends and conclusions presented here might differ if the study had been conducted with broader inclusion of experts from different countries of origin (Nuñez et al., 2019). This may suggest that future analysis of drivers should be undertaken by involving representatives from a wider selection of countries worldwide, so to fine‐tune the result at a broader scale. Similarly, future scenarios assessed at the regional or continental scale may be used to inform policy and management measures to be undertaken at the respective scales.

## 5 CONCLUSIONS

Understanding how and why the impacts of invasive alien species might change in the future is a daunting task that has so far defied the development of quantitative scenarios and models (Lenzner et al., 2019). We suggest that expert‐based assessments provide a valuable tool to support quantitative assessments and may help identify emerging threats and directions for future research. We demonstrated that, based on expert knowledge, there is a high risk of increased potential future impacts of biological invasions due to many drivers, especially increased trade and transport (Hulme, 2009), climate change (Walther et al., 2009) and socio‐economic change (Pyšek et al., 2010). Our assessment can be used to develop recommendations for policy‐makers and environmental managers. In particular, our findings provide a scientific basis for the prioritization of actions to mitigate potential future impacts of biological invasions in the context of the Post‐2020 Framework of the Convention on Biological Diversity (CBD, 2020) and the United Nations 2030 Agenda for Sustainable Development (United Nations, 2016). Most importantly, our study provides expert‐derived benchmarks for thresholds of major impacts in different socioecological contexts, identifies which drivers are most likely to cause substantial impacts and identifies potential options under best‐case scenarios to reduce potential future impacts of biological invasions.

## AUTHOR CONTRIBUTION

F.E., N.R‐.P., Be.L. and W.R. organized the workshop that formed the basis for this manuscript. F.E. conceived the ideas and designed the study, with input from several other authors. All authors (except B.J.M.) completed the survey. Be.L. led the analysis with help from F.E., S.D. and B.J.M., F.E. and Be.L. wrote the paper with major inputs from S.D. All authors commented on the manuscript.

## Supporting information

Supplementary Material

Supplementary Material

## Data Availability

Data will be shared upon reasonable request to the authors. Please note that the survey responses will only be shared in an anonymized fashion that does not allow drawing conclusions about the respondents’ identity.
