# Supplementary material for: Drivers of future alien species impacts: An expert‐based assessment
Source: Glob Chang Biol. 2020 Jul 14;26(9):4880–93. doi: 10.1111/gcb.15199 (PMC7496498; doi:10.1111/gcb.15199)
Supplement: Supplementary file 1 — Supplementary Material [file GCB-26-4880-s001.docx]

## Drivers of future alien species impacts: an expert-based assessment

Franz Essl, Bernd Lenzner, Sven Bacher, Sarah Bailey, Cesar Capinha, Curtis Daehler, Stefan Dullinger, Piero Genovesi, Cang Hui, Philip E. Hulme, Jonathan M. Jeschke, Stelios Katsanevakis, Ingolf Kühn, Brian Leung, Andrew Liebhold, Chunlong Liu, Hugh J. MacIsaac, Laura A. Meyerson, Martin A. Nuñez, Aníbal Pauchard, Petr Pyšek, Wolfgang Rabitsch, David M. Richardson, Helen E. Roy, Gregory M. Ruiz, James C. Russell, Nathan J. Sanders, Dov F. Sax, Riccardo Scalera, Hanno Seebens, Michael Springborn, Anna Turbelin, Mark van Kleunen, Betsy von Holle, Marten Winter, Rafael D. Zenni, Brady J. Mattsson & Nuria Roura-Pascual

**Supplementary Material**

*Supplementary Material 1*. Survey instructions (including list of 15 drivers relevant for biological invasions, descriptions of best respectively worst case-scenarios) sent to the survey respondents.

**An expert-based assessment of the drivers of future biological invasions in contrasting environmental, taxonomic and socio-economic contexts**

***Goal: An assessment of the importance and uncertainty of how particular drivers may affect biological invasions in contrasting contexts until 2050, based on the knowledge of experts of biological invasions.***

Approach: The assessment is being done by a group of scientists with expertise in biological invasions. The core team of the assessors participated in an invasion scenario workshop held in Vienna, Austria in October 2016, and in the subsequent development of invasion scenarios, i.e. the development of plausible scenarios representing how biological invasions might develop under contrasting socio-economic and societal conditions until the mid-21^st^ century (Roura-Pascual et al. in prep.).

The list of drivers relevant for biological invasions was compiled as part of preparing for the above-mentioned scenarios workshop. From a long list of putatively relevant drivers, the team (ca. 20 scientists with background in invasion ecology, global change science etc.) has identified and pre-selected a set of 15 direct drivers (sensu Ferrier et al. 2016) that have been considered most relevant for determining future spread of invasive species.

Task: We have incorporated the survey into an Excel-Table. This has proven to be most practical and the preferred option in the pre-test of the survey. First, please review the list of 15 pre-selected key drivers (Supplementary Table 1) for shaping biological invasions until the mid-21^st^ century (2050) in particular contexts (e.g. ecosystem type, taxonomic group) (Table A of the Excel-file of the survey, cf. Supplementary Material 2), and assess the importance and uncertainty for each. Second, please provide some information on your background and expertise (Table B of the Excel-file of the survey, cf. Supplementary Material 2). When quantifying the importance and uncertainty for each driver, please use the following approach:

- The response variable we ask you to assess, i.e. biological invasions, is defined as the change in negative impacts of alien species in the specific environmental, taxonomic or socio-economic context. Imagine that each of the drivers in different contexts in Table A of the survey may have an influence on the future development of biological invasions. We ask you to assess what would constitute an increase in major negative impacts of alien species on i) biodiversity and ii) human livelihoods. Please provide these two ratings as an increase in % compared to current impacts of alien species. In a next step, we ask you to assess the importance of each driver by defining it as probability (using 5 classes) that increases in impacts of alien species in the specific context will by 2050 exceed the thresholds you have defined above for major impacts on i) biodiversity and ii) human livelihoods, holding all other drivers at their current levels.
- We suggest to concentrating in the assessment on the impacts caused by alien species. However, we recognize that estimated changes in number and abundance of alien species in different contexts may also serve as a useful proxy for changes in impacts caused by alien species.
- For each context and hypothesized driver, please fill in each cell in the Excel file (see instructions below). Assess the importance of the driver and also the uncertainty of the assessment (based on your expertise) by using a five-step numerical approach. We ask you to provide your assessments for a best and worst case scenario (for definitions of best / worst case scenarios, see Supplementary Table 1).

**Instructions for filling in the Table A in the Excel-file of the survey**

Please carefully read the instructions below before you fill in Table A in the Excel-file of the survey. They are intended to reduce (epistemic and linguistic) uncertainties by providing definitions of relevant key terms, and guidance for interpreting them.

*Definition of major impact on biodiversity and human livelihoods:*

The increase in alien species impact compared to the status quo that would cause a major negative impact on biodiversity and human livelihoods in a specific context.

As “major negative impact” on biodiversity we define any substantial “*changes in community composition*” such as local extinction of at least one native species, severe decline of several native species, and substantial changes in ecosystem properties (structure, complexity, functioning) (Blackburn et al. 2014, modified).

As “major negative impact” on human livelihoods we define any substantial “*impact on peoples’ well-being that leads to a local decline or abandonment of human activity*”; examples would include the changes in planting of a specific crop species, changes in silvicultural practices, or changes in specific recreational activities such as fishing, hiking) *due to the impacts* (e.g. management costs, yield losses, impacts on human health) *of alien species* (Bacher et al. 2017, modified). Major negative impact does not necessarily imply the change of activities from the entire region assessed, but refers to the typical spatial scale over which social communities in the region are characterized (e.g. in our case, we suggest that the appropriate scale should be human settlements, professions or other important activities (e.g. recreational or traditional activities)).

Please provide these two ratings as an increase of impacts of alien species (in %) compared to the status quo in the specific context.

Example: If you believe that a major negative impact on the biodiversity in terrestrial habitats would result from an increase of 30% in alien species impacts, please fill in “30”.

If you believe that a major negative impact on human livelihoods in terrestrial habitats would result from an increase of 50% in alien species impacts, please fill in “50”.

*Definition of the categories on assessment uncertainty: (in 20%-steps)*

Assess the uncertainty of your assessment that the change of a driver will cause major impacts on biodiversity or human livelihoods by using a five-step numerical approach.

1 = extremely uncertain (0-20% certain)

2 = moderately uncertain (21-40 % certain)

3 = medium certain (41-60 % certain)

4 = highly certain (61-80 % certain)

5 = extremely certain (81-100% certain)

In the context of this assessment, we are interested in the assumed uncertainty based on the expertise and knowledge of the assessor (cf. Mastandrea et al. 2010). This will allow us to perform analyses of the assessments afterwards. So, please strictly use these values.

We encourage you to provide your estimates even in situations of high uncertainty. In situations where you believe you have insufficient expertise to respond to a particular question, please provide “NA“ (= Not Assessed) as answer. A few drivers (such as “Ocean acidification”) are not relevant in all contexts, and so no answer is needed in such cases. This is already indicated in the survey with “NR”(= Not Relevant) and yellow colour.

*Definition of probability: (in 20%-steps)*

For each driver and environmental, taxonomic and socio-economic context combination, we ask you the following: Please assess the importance of each driver by defining it as the probability (in %) that impacts of alien species in the specific context will by 2050 exceed the thresholds you have defined above for causing major impacts on i) biodiversity and ii) human livelihoods, holding all other drivers at their current levels.

Again, we ask you to use a five-step numerical approach.

1 = extremely uncertain (0-20% certain)

2 = moderately uncertain (21-40 % certain)

3 = medium certain (41-60 % certain)

4 = highly certain (61-80 % certain)

5 = extremely certain (81-100% certain)

Example: If you believe that the consequences of climate change on alien species impacts will cross the threshold for major negative impacts on biodiversity in terrestrial environments with a 30% probability by 2050, please fill in “2”.

If you believe that the consequences of climate change on alien species impacts will cause to cross the threshold for major negative impacts on human livelihoods in terrestrial environments with a 70% probability by 2050, please fill in “4”.

NB 1: Please assess each driver in isolation and disregard any potential interactions with other drivers.

NB 2: In a few cases, a driver will be not relevant for a specific context (e.g. ocean acidification for terrestrial environments). In these cases, please enter “NR” in the respective field.

*Supplementary Table 1*: The 15 direct drivers considered to be most relevant for biological invasions until 2050. Main attributes of each driver, short descriptions of the drivers, best case- and worst case-scenarios, and the relevant sources for these scenarios are listed.

| **Driver** | **Attributes** | **Definition / Impacts on invasions** | **Best case scenario** | **Worst case scenario** | **Scientific evidence** |
| --- | --- | --- | --- | --- | --- |
| Global abiotic environmental change | | | | | |
| Climate change | Temperature increase, precipitation changes, increase in climatic extreme events | Climate change is likely to change mean temperatures, change precipitation patterns etc. and increase the frequency, magnitude and distribution of extreme events, causing disturbances that may create opportunities for alien species. These changes are likely to interact synergistically with biological invasions, although substantial variation exists among taxa and geographic regions. | The IPCC- RCP 2.6 scenario that likely results in an average increase of the global mean temperature by around 1.0°C until 2050 (compared to mid-20^th^ century levels) | The IPCC-RCP 8.5 scenario that likely results in an average increase of the global mean temperature by around 2.5°C until 2050 (compared to mid-20^th^ century levels) | Walther et al. (2009), Bellard et al. (2013), Chown et al. (2015) |
| Ocean acidification | Increasing acidity of Ocean waters due to rising atmospheric CO_2_-levels | Increasing CO_2_-levels will increase ocean acidification, thereby affecting water chemistry and native biota and likely facilitating biological invasions | The IPCC - RCP 2.6 scenario likely results in a decrease of the sea water pH values from 8.11 to 8.05. | The IPCC - RCP 8.5 scenario likely results in a decrease of the sea water pH values from 8.11 to 7.80. | Hoegh-Goldberg et al. (2007) |
| Eutrophication and pollution | Deposition of pollutants and nutrients | Anthropogenic input of pollutants and nutrients via fertilization, run off and atmospheric deposition affect many ecosystems. Nutrient input often promotes opportunistic species, which are often invasive alien species (IAS). | Application and deposition of nutrients (nitrogen, phosphorus), pesticides and pollutants remains stable in most regions, leading to much reduced impacts such as eutrophication, anoxia in coastal seas and freshwaters, and accumulation of pesticides and pollutants. | Application and deposition of nutrients (nitrogen, phosphorus), pesticides and pollutants is increasing substantially causing widespread impacts such as eutrophication, anoxia in coastal seas and freshwaters, and accumulation of pesticides and pollutants. | Galloway et al. (2004), Bobbink et al. (2010) |
| Global biotic environmental change | | | | | |
| Biodiversity loss and degradation | Ecosystem loss and degradation, overharvesting, defaunation, trophic downgrading, biological meltdown | Downgrading and loss of (near-) natural ecosystems, loss of species and functional groups, and positive feedbacks (facilitation, invasional meltdown) may promote opportunistic alien species | Biodiversity and ecosystem services will become highly valued. Spending for conservation will increase substantially and halting biodiversity decline will become a key political priority. | Biodiversity and ecosystem services are not valued, neither socially nor economically. Spending for conservation will decline. The on-going loss of biodiversity will continue and even increase substantially in many regions of the world. | Simberloff (2006), Estes et al. (2011), Dirzo et al. (2014) |
| Socio-economic activities | | | | | |
| Trade and transport | Changes in global trade (modes, routes, goods, volumes, velocity) | Key features of trade and transport such as the goods that are transported, the volumes that are traded, the means and velocity of transport, and the routes of transport are likely to change in the future. New modes of trade (e.g. via internet) that are more difficult to regulate may become more relevant for biological invasions. Trade includes also specific high-risk pathways such as pet and horticultural trade, wood products, ballast water and biofouling, and the emergence of new trade routes that are becoming accessible due to climate change (Arctic shipping routes) or economic interests (Suez Canal and Panama Canal extension, Nicaragua Canal). | Global trade volumes will roughly double between 2015 and 2050 (OECD/ITF 2017). | Global trade volumes will increase by more than 4-fold between 2015 and 2050 (OECD/ITF 2017). | Levine & D’Antonio (2003), Niimi (2004), Niinemets & Penuelas (2008), Liebhold et al. (2012), Seebens et al. (2013, 2015), Williams et al. (2013), Brockerhoff et al. (2014), Leung et al. (2014), Humair et al. (2015) |
| Land-use and land use-change | Land-use and land-use changes associated with agriculture, forestry, aquaculture, climate change adaptation and mitigation (e.g. bioenergy production), and expansion of settlements and infrastructure | Demand to feed, clothe, host, etc. and to provide new materials (e.g. for bioenergy production) will likely increase. The resulting changes in land-use (incl. the intentional use of IAS) and land-use intensity may cause losses of ecosystems, degradation of used ecosystems, increase fragmentation and disturbance of ecosystems, and alter resource dynamics; all these are factors that may favour biological invasions and reduce biotic resistance. | The IPCC - RCP 2.6 scenario assumes a decrease of cultivated land (due to intensification, changes in human diets etc.) and an increase in natural land in 2050 compared to 2000. | The IPCC - RCP 8.5 scenario assumes a substantial increase by 185 million ha of cultivated land between 2000 and 2050. | With (2004), Riley et al. (2005), Didham et al. (2007), Minor et al. (2009), Früh et al. (2012) Trentanovi et al. (2013), Erb et al. (2016), Newbold et al. (2015), Paterson et al. (2012) |
| Socio-economic development | Change of socio-economic-activities that correlate to resource and energy uses, human mobility, intensity of land use etc. | The level of socio-economic activities (as measured by per capita GDP or similar metrics) is correlated with a wide range of changes of the environment (e.g. resource and energy uses, human mobility, land use) that may be relevant for facilitating biological invasions. | The global material footprint increases to 132 billion metric tons (equivalent to a rise by 75% over the 2015 levels) (IRP 2017). | The global material footprint rises to over 184 billion metric tons (=more than double the 2015 levels) (IRP 2017). | Pyšek et al. (2010), Essl et al. (2011), Gallardo (2014) |
| Human population size and migration | Size of the human population, its spatial distribution and migration | Changes in the size and distribution of human populations and migration of humans may influence biological invasions via a range of correlated relevant impacts. | Population size increases to 8.5 billion by 2050. Global fertility is low and migration stays similar to today (according to SSP1; KC & Lutz 2014). | Population size reaches 10.0 billion by 2050. Fertility remains high in developing countries with high mortality rates. International migration is overall low (because of an emphasis on security and borders) (according to SSP3; KC & Lutz 2014). | Pyšek et al. (2010), Essl et al. (2011), Spear et al. (2013) |
| Societal awareness, values, lifestyle | | | | | |
| Recreation and tourism | Recreation activities and (inter)national tourism | (Outdoor) recreation (incl. gardening, hunting, fishing, hiking) and tourism may impact on biological invasions in a range of different ways. | The United Nations World Tourism Organization (UNWTO 2011) projects in a slow growth scenario an increase in global tourist arrivals by 2.2 billion until until 2050 (compared to 1.2 billion in 2010). | The United Nations World Tourism Organization (UNWTO 2011) projects in a “falling transport costs” scenario an increase in global tourist arrivals by 3.2 billion until 2050 (compared to 1.2 billion in 2010). | Anderson et al. (2015), Hughes et al. (2015) |
| Societal awareness and values | Societal awareness and values | The awareness and values of the citizens, stakeholders, business, NGOs and politicians towards biological invasions is important for establishing and implementing IAS policies and management. Includes also the views of people who are opposing actions on IAS on ethical grounds (e.g. animal-right movements) or because they consider it unwarranted. | There is a high environmental awareness among citizens, which empower people to participate effectively in democratic changes towards a better environment for all (e.g. reduce consumption and responsible buying). Planetary consciousness (interdependence and interconnection of all humans and the earth). | There is no environmental awareness among citizens. People act individually and unconsciously, without caring for the environment. Selfish society. Consumption rates increase. | Blackburn et al. (2010), Roura-Pascual et al. (2011), Glen et al. (2013), Tollington et al. (2015) |
| Communication and outreach | Media, social media, personal communication | The way, tone and intensity of communication on biological invasions with(in) the wider public and decision makers may influence the public perception of and action on IAS. | There is intense and appropriate communication on IAS issues resulting in a high awareness of the issue of biological invasions. | There is little and inappropriate (i.e. biased, cf. invasion denial) communication on IAS issues resulting in a low awareness of the issue of biological invasions. | García-Llorente et al. (2008), Selge et al. (2011), Brunel (2014) |
| Science, innovation and technology | | | | | |
| IAS science | Scientific research | Scientific research on IAS may improve the understanding of the invasion process of IAS, improve management techniques, increase data availability on alien species etc. Further, research priorities may be more or less relevant for IAS management and policy. | Scientific research on the causes, processes and consequences of biological invasions are a priority and lead to many new insights for IAS management. | Scientific research on the causes, processes and consequences of biological invasions are not considered important and there are few new insights for IAS management. | Caffrey et al. (2014), Hulme (2015), Lovett et al. (2016) |
| Technology and innovation | Technological progress and innovation | The general level of innovation, and the extent to which new technologies are introduced, accepted and become widely applied. | High rates of technological progress and innovation. No limits in resources and development capacity. Technological progress leads to profound socio-economic and societal changes. | Low rates of technological progress and innovation. Limits in resources and development capacity. Technological progress has relatively moderate socio-economic and societal effects. | Rocchini et al. (2015), Thomsen & Willersley (2015) |
| Societal response to IAS: legislation, policy, cooperation, management | | | | | |
| Cooperation, legislation and agreements | Institutional and political cooperation, global to national legislation and agreements | The level of political and institutional cooperation (within and between nations) and the ensuing legislation and agreements on biosecurity and biological invasions, their relationship with other relevant topics (e.g. trade agreements), and the level of implementing these policies. | Sustainability is high on the political agenda. The Sustainable Development Goals of the 2030 Agenda for Sustainable Development (UN 2015) are reached. | Sustainability is no priority on political agendas. The Sustainable Development Goals of the 2030 Agenda for Sustainable Development (UN 2015) are not reached and have no impact. | Perrings et al. (2010), Eschen et al. (2015), Tollington et al. (2015), Lovett et al. (2016) |
| IAS management | Level and comprehensiveness of IAS management | The comprehensiveness and level of implementation of IAS management, and the available resources and institutional capacities may be important for the level of biological invasions. | IAS management is taken very seriously on all scales. Existing and new regulations cover all relevant aspects of biological invasions, and there is a very good level of implementing IAS regulations. | IAS management is a low priority on all scales. Many relevant aspects of biological invasions are not covered by regulations, and implementation of existing IAS regulations is poor. | Roura-Pascual et al. (2011), Hajek et al. (2016) |

**Instructions for filling in the Table B in the Excel-file of the survey**

When you have filled in Table A of the Excel-file of the survey, we would like to ask you to fill in Table B in the Excel-file of the survey and to provide some additional personal and professional information (see Supplementary Table 2). The more detailed instructions have been incorporated into Table B in the Excel-file (Supplementary Material 2).

*Supplementary Table 2.* Short descriptions of the environmental, taxonomic and socio-economic contexts asked for assessment in Table B of the Excel-file of the survey.

| **Category** | **Definition** |
| --- | --- |
| ***Realms*** |  |
| Terrestrial | Terrestrial habitats (including on continents and islands) |
| Freshwater | Freshwater habitats |
| Marine | Habitats of the coasts and seas |
| ***Socio-economic status*** |  |
| Developed countries | Socio-economically highly developed countries (i.e. OECD countries) |
| Emerging and transition countries | Socio-economically rapidly developing emerging economies (e.g. China, India, Vietnam, Brazil, Chile, Argentina, South Africa), economies in transition (e.g. former communist countries such as Russia, Poland, Ukraine), and middle income countries (e.g. many countries of North Africa and Latin America). |
| Developing countries | Socio-economically poor countries with mostly slow rates of economic growth (many sub-Saharan African countries, several countries in SE Asia and Latin America) |
| ***Zonobiomes*** | ***According the approach of Walter 2002*** |
| Tropical | Tropical diurnal and tropical savanna zonobiomes |
| Subtropical | Subtropical arid and Mediterranean zonobiomes |
| Temperate | Warm temperate humid, nemoral and continental zonobiomes |
| Polar | Boreal and arctic zonobiomes |
| ***Taxonomic groups*** |  |
| Plants | All plant groups (algae to vascular plants) |
| Vertebrates | Fishes, amphibians, reptiles, birds, mammals |
| Invertebrates | Such as insects, arachnids, crustaceans, molluscs, echinoderms, jellyfish, … |
| Microorganisms | Uni-cellular organisms (incl. pathogens) |

***References for Supplementary Material 1***

Blackburn, TM, et al. (2010) Dying for conservation: eradicating invasive alien species in the face of opposition. Animal Conservation 13, 227-228.

Bringezu S et al. (2017) A Report of the International Resource Panel. United Nations Environment Programme. Nairobi, Kenya.

Brunel S (2014) How to communicate on pests and invasive alien plants? Conclusions of the EPPO/CoE/IUCN-ISSG/DGAV/UC/ESAC Workshop. EPPO Bulletin 44, 205–211.

Caffrey et al. (2014) Tackling invasive alien species in Europe: the Top 20 issues. Management of Biological Invasions 5, 1-20.

Chiron F et al. (2010) Behind the Iron Curtain: Socio-economic and political factors shaped exotic bird introductions into Europe. Biol. Conserv. 143: 351-356.

Chown, S.L., Hodgins, K.A., Griffin, P.C., Oakeshott, J.G., Byrne, M., Hoffmann, A.A. (2015) Biological invasions, climate change and genomics. Evolutionary Applications 8: 23-46.

Didham, R.K., Tylianakis, J.M., Gemmell, N.J., Rand, T.A. & Ewers, R.M. (2007) Interactive effects of habitat modification and species invasion on native species decline. Trends Ecol. Evol. 22, 489–496.

Dirzo R et al. (2014) Defaunation in the Anthropocene. Science 345: 401-406.

Erb KH et al. (2016) Exploring the biophysical option space for feeding the world without deforestation. Nature Communications, 7, doi:10.1038/ncomms11382.

Eschen R et al. (2015) International variation in phytosanitary legislation and regulations governing importation of plants for planting. Environ Sci Pol 51: 228–237.

Estes JA et al. (2011) Trophic downgrading of planet Earth. Science 333: 301-306.

Ferrier S, Ninan KS, Leadley P, Alkemade R, Kolomytsev G, Moraes M, Mohammed EY & Trisurat Y (2016) Overview and vision. In: IPBES: Methodological assessment of scenarios and models of biodiversity and ecosystem services. Secretariat of the Intergovernmental Platform for Biodiversity and Ecosystem Services, Bonn, Germany.

Früh D et al. (2012) Physicochemical and morphological degradation of stream and river habitats increases invasion risk. Biol. Invasions 14: 2243-2253.

Gallardo B (2014) Europe’s top 10 invasive species: relative importance of climatic, habitat and socio-economic factors. Ethology, Ecology and Evolution 26: 130-151.

Galloway JN, Dentener FJ, Capome DG, Boyer EW, Howarth RW, Seitzinger SP, Asner GP, Cleveland CC, Green PA, Holland EA, Karl DM, Michaels AF, Porter JH, Townsend AR & Vöosmarty CJ (2004) Nitrogen cycles: past, present and future. Biogeochemistry, 70, 153-226.

García-Llorente, M., Martín-López, B., González, J.A., Alcorlo, P., Montes, C. (2008) Social perceptions of the impacts and benefits of invasive alien species: Implications for management. Biological Conservation 141, 2969–2983.

Glen et al. (2013) Eradicating multiple invasive species on inhabited islands: the next big step in island restoration? Biological Invasions DOI 10.1007/s10530-013-0495-y

Hajek et al. (2016) Exotic biological control agents: a solution or contribution to arthropod invasions? Biol Invasions. doi:10.1007/s10530-016-1075-8.

Hoegh-Goldberg O, Mumby PJ, Hooten AJ, et al. (2007) Coral reefs under rapid climate change and ocean acidification. Science, 318, 1737-1742.

Hughes KA et al. (2015) Biological invasions in terrestrial Antarctica: what is the current status and can we respond? Biodivers Conserv 24: 1031-1055.

IUCN (2013) Guidelines for Reintroductions and Other Conservation Translocations. IUCN SSC, Gland, Switzerland, ed. 1.0.

Liebhold AM et al. (2013) Live plant imports: the major pathway for forest insects and pathogen invasion of the US. Front. Ecol. Environ. 10: 135-143.

Levine JM, D’Antonio CM (2003) Forecasting biological invasions with increasing international trade. Conserv Biol 17: 322-326.

Lovett G, Weiss M, Liebhold AM, Holmes TP, Leung B, et al. (2016) Nonnative forest insects and pathogens in the United States: Impacts and policy options. Ecological Applications, 26, 1437-1455.

Mastrandrea, M.D., C.B. Field, T.F. Stocker, O. Edenhofer, K.L. Ebi, D.J. Frame, H. Held, E. Kriegler, K.J. Mach, P.R. Matschoss, G.-K. Plattner, G.W. Yohe, F.W. Zwiers (2010) Guidance Note for Lead Authors of the IPCC Fifth Assessment Report on Consistent Treatment of Uncertainties. Intergovernmental Panel on Climate Change (IPCC). www.ipcc.ch.

Minor ES et al. (2009) The role of landscape connectivity in assembling exotic plant communities: a network analysis. Ecology 90: 1802-1809.

Newbold T et al. (2015) Global effects of land use on local terrestrial biodiversity. Nature 520: 45-50.

Perrings, C. et al. (2010): International cooperation in the solution to trade-related invasive species risks. Ann. NY Acad. Sci. 1195: 198-212.

Riley SP et al. (2005) Effects of urbanization on the distribution and abundance of amphibians and invasive species in southern California streams. Conserv. Biol. 19: 1894-1907.

Rocchini, D., Andreo, V., Forster, M., Garzon-Lopez, C. X., Gutierrez, A. P., Gillespie, T. W., et al. (2015) Potential of remote sensing to predict species invasions: a modelling perspective. Progr. Phys. Geogr. 39, 283–309.

Roura-Pascual N, Richardson DM, et al. (2011) Managing biological invasions: charting courses to desirable futures in the Cape Floristic Region. Regional Environmental Change 11:311-320.

Rowland, E.R., Cross, M.S., Hartmann, H. (2014) Considering Multiple Futures: Scenario Planning To Address Uncertainty in Natural Resource Conservation. Washington, DC: US Fish and Wildlife Service.

Seebens et al. (2013) The risk of marine bioinvasion caused by global shipping. Ecol Lett 16: 782-790.

Selge, S., Fischer, A. & van der Wal, R. (2011) Public and professional views on invasive non-native species – A qualitative social scientific investigation. Biological Conservation. 144: 3089–3097.

Simberloff D (2006) Invasional meltdown 6 years later: important phenomenon, unfortunate metaphor, or both? Ecol. Lett. 9: 912-919.

Swan KD et al. (2016) Managing Marine Biodiversity: The Rising Diversity and Prevalence of Marine Conservation Translocations. Conserv. Lett. DOI: 10.1111/conl.12217

Thomsen, P. F., Willerslev, E. (2015) Environmental DNA - An emerging tool in conservation for monitoring past and present biodiversity. Biological Conservation 183, 4-18.

Tollington, S., Turbé, A., Rabitsch, W., Groombridge, J., Roy, H., Scalera, R., Essl, F. & Shwartz, A. (2015) Making the EU legislation on invasive species a conservation success. Conservation Letters, doi 10.1111/conl.12214.

Trentanovi, G., et al. (2013) Biotic homogenization at the community scale: disentangling the roles of urbanization and plant invasion. Diversity Distrib. 19: 738-748.

Walter H (2002) Walter's vegetation of the earth: the ecological systems of the geo-biosphere. Springer, Berlin.

Walther, G.-R., A. Roques, P. E. Hulme, M. T. Sykes, P. Pyšek, I. Kühn, M. Zobel et al. (2009) Alien species in a warmer world: risks and opportunities. Trends in Ecology and Evolution 24:686–693.

Williams, SL et al. (2013) Managing multiple vectors for marine invasions in an increasingly connected world. BioScience 63: 952-966.

With K (2004) Assessing the risk of invasive spread in fragmented landscapes. Risk Anal 24: 803-805.

*Supplementary Material 2*. Survey sent to the survey respondents.

This file is submitted as an extra-file.

*Supplementary Material 3. Expertise and backgrounds of the respondents*

We asked the respondents to provide a self-assessment of their expertise regarding their knowledge on different taxonomic groups, realms and biomes, as well as geographical regions, socio-economic context and research focus (Supplementary Table 3). The highest expertise (selecting high or very high expertise categories) was concentrated in Europe (58% of the respondents) and North America (47%) followed by South America (17%), the Pacific Islands (17%), Australia (14%), Africa (14%) and Asia (11%).

Among biomes, expertise on temperate biomes (86%) was reported by far most frequently, followed by subtropical (19%), polar (9%) and tropical (6%). In terms of the socio-economic context, most respondents considered themselves as experts within developed countries (78%), followed by countries with emerging economies (19%) and developing countries (3%).

Taxonomic expertise varied among respondents as follows: plants (61%), invertebrates (47%), vertebrates (44%) and microorganisms (14%). Expertise also varied by realm, with most knowledge in the terrestrial (78%) realm followed by freshwater (36%) and marine (19%). Finally, most experts work within the field of pure invasion science (86%), followed by invasion alien species policy (50%) and applied invasion science (47%).

Among the 36 respondents that fully completed the survey, 30 were male and 6 female. The age structure of the respondents was: 3 respondents < 30 years; 7 respondents between 30 and 40 years; 10 respondents between 40 and 50 years; and 6 respondents > 50 years. Ten respondents did not provide information on their age.

*Supplementary Table 3*: Self-ascribed expertise of the 36 participating experts for the different contexts. Levels of expertise were given on a five-point Likert scale. As experts could choose multiple categories all answers were standardized between 0 and 1.


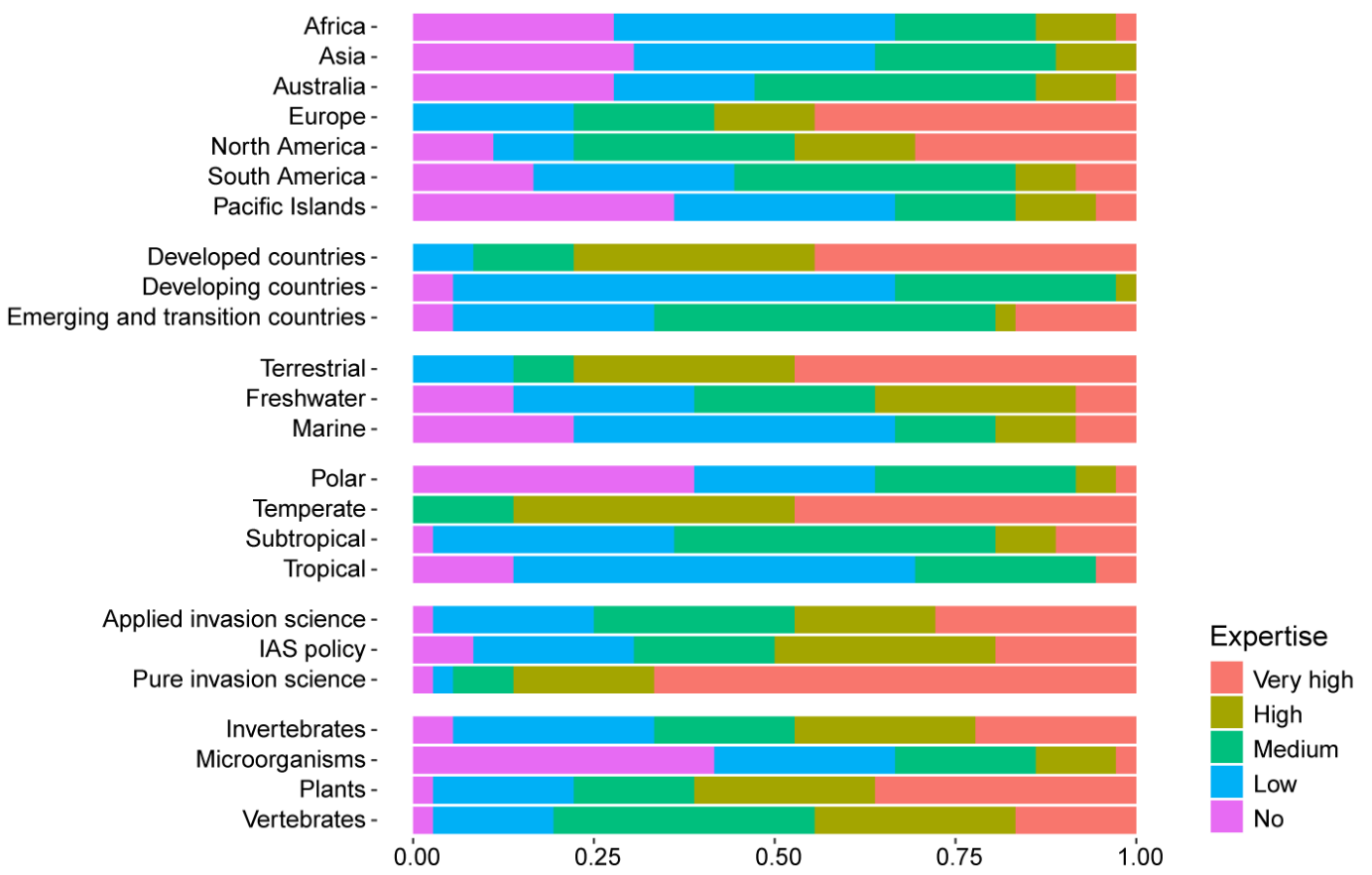


*Supplementary Material 4*. Summary of the answers given for the threshold of major impacts on biodiversity across different contexts. Reported are the median value of all answers as well as the 90% quantiles.

| **System** | **Median** | **5% quantile** | **95% quantile** |
| --- | --- | --- | --- |
| Polar | 30 | 5 | 225 |
| Temperate | 30 | 10 | 77.5 |
| Subtropical | 22.5 | 10 | 62.5 |
| Tropical | 30 | 10 | 60 |
| Invertebrates | 22.5 | 10 | 62.5 |
| Microorganisms | 20 | 3.75 | 70 |
| Vertebrates | 20 | 3.75 | 42.5 |
| Plants | 30 | 7.5 | 70 |
| Freshwater | 20 | 8.75 | 50 |
| Marine | 30 | 10 | 90 |
| Terrestrial | 20 | 7.5 | 62.5 |
| Developed countries | 30 | 13.75 | 75 |
| Developing countries | 30 | 3.75 | 80 |
| Countries with emerging economies | 20 | 8.75 | 62.5 |

*Supplementary Material 5*. Results of the non-parametric, pairwise Komolgorov-Smirnov test comparing density distribution across different A) zonobiomes, B) taxonomic groups, C) realms and D) socio-economic status. Bold values indicate significant p-values < 0.05. Significant results indicate that the distributions are significantly different from each other.

| **A)** |  |  |  |  |  |  |
| --- | --- | --- | --- | --- | --- | --- |
|  | **Temperate** | | **Subtropical** | | **Tropical** | |
|  | *D* | *p-value* | *D* | *p-value* | *D* | *p-value* |
| **Polar** | 0.25 | 0.211 | 0.25 | 0.211 | 0.19 | 0.504 |
| **Temperate** |  |  | 0.083 | 1 | 0.19 | 0.504 |
| **Subtropical** |  |  |  |  | 0.19 | 0.504 |
|  |  |  |  |  |  |  |
| **B)** |  |  |  |  |  |  |
|  | **Microorganisms** | | **Vertebrates** | | **Plants** | |
|  | *D* | *p-value* | *D* | *p-value* | *D* | *p-value* |
| **Invertebrates** | 0.17 | 0.699 | 0.25 | 0.211 | 0.14 | 0.878 |
| **Microorganisms** |  |  | 0.14 | 0.878 | 0.28 | 0.124 |
| **Vertebrates** |  |  |  |  | **0.36** | **0.018** |
|  |  |  |  |  |  |  |
| **C)** |  |  |  |  |  |  |
|  | **Marine** | | **Terrestrial** | |  |  |
|  | *D* | *p-value* | *D* | *p-value* |  |  |
| **Freshwater** | **0.36** | **0.018** | 0.14 | 0.878 |  |  |
| **Marine** |  |  | 0.22 | 0.336 |  |  |
|  |  |  |  |  |  |  |
|  |  |  |  |  |  |  |
| **D)** |  |  |  |  |  |  |
|  | **Developing countries** | | **Emerging countries** | |  |  |
|  | *D* | *p-value* | *D* | *p-value* |  |  |
| **Developed countries** | 0.22 | 0.336 | 0.19 | 0.504 |  |  |
| **Developing countries** |  |  | 0.17 | 0.699 |  |  |
|  |  |  |  |  |  |  |

*Supplementary Material 6.* Estimates from ordinal logistic regression for the likelihood that 15 major drivers of biological invasions will cause major environmental impacts by 2050 in best-case and worst-case scenarios. Given are the results for the best (A) and worst (B) case scenarios for all drivers in the relevant socio-ecological context. Significance levels are defined as following: p < 0.001 = ***; p < 0.01 = **; p < 0.05 = *. Bold drivers are those with a p value < 0.05.

1. Best-case scenario

| **Driver** | **Socio-ecol. context** | **Scenario** | **estimate** | **conf.low** | **conf.high** | **p.value** |  |
| --- | --- | --- | --- | --- | --- | --- | --- |
|  |  |  |  |  |  |  |  |
| Biodiversity loss and degradation | Polar | best | 0.62 | 0.41 | 0.80 | 0.271 |  |
| **Climate change** | **Polar** | **best** | **0.91** | **0.78** | **0.97** | **0.000** | ******* |
| Communication and outreach | Polar | best | 0.40 | 0.21 | 0.61 | 0.339 |  |
| Cooperation, legislation and agreements | Polar | best | 0.56 | 0.35 | 0.75 | 0.608 |  |
| Eutrophication and pollution | Polar | best | 0.56 | 0.35 | 0.75 | 0.608 |  |
| Human population size and migration | Polar | best | 0.49 | 0.29 | 0.70 | 0.941 |  |
| IAS management | Polar | best | 0.36 | 0.19 | 0.58 | 0.211 |  |
| IAS science | Polar | best | 0.49 | 0.29 | 0.70 | 0.941 |  |
| Land-use and land use-change | Polar | best | 0.59 | 0.37 | 0.77 | 0.420 |  |
| Ocean acidification | Polar | best | 0.65 | 0.44 | 0.82 | 0.163 |  |
| **Recreation and tourism** | **Polar** | **best** | **0.71** | **0.50** | **0.86** | **0.048** | ***** |
| Societal awareness and values | Polar | best | 0.43 | 0.24 | 0.64 | 0.507 |  |
| **Socio-economic d** | **Polar** | **best** | **0.77** | **0.57** | **0.89** | **0.010** | ***** |
| Technology and innovation | Polar | best | 0.40 | 0.21 | 0.61 | 0.338 |  |
| **Trade and transport** | **Polar** | **best** | **0.87** | **0.71** | **0.95** | **0.000** | ******* |
|  |  |  |  |  |  |  |  |
| Biodiversity loss and degradation | Subtropical | best | 0.68 | 0.47 | 0.84 | 0.091 |  |
| **Climate change** | **Subtropical** | **best** | **0.82** | **0.64** | **0.92** | **0.002** | ****** |
| Communication and outreach | Subtropical | best | 0.49 | 0.29 | 0.70 | 0.941 |  |
| Cooperation, legislation and agreements | Subtropical | best | 0.59 | 0.37 | 0.77 | 0.420 |  |
| **Eutrophication and pollution** | **Subtropical** | **best** | **0.71** | **0.50** | **0.86** | **0.048** | ***** |
| **Human population size and migration** | **Subtropical** | **best** | **0.82** | **0.64** | **0.92** | **0.002** | ****** |
| IAS management | Subtropical | best | 0.49 | 0.29 | 0.70 | 0.941 |  |
| IAS science | Subtropical | best | 0.49 | 0.29 | 0.70 | 0.941 |  |
| **Land-use and land use-change** | **Subtropical** | **best** | **0.71** | **0.50** | **0.86** | **0.048** | ***** |
| Ocean acidification | Subtropical | best | 0.65 | 0.44 | 0.82 | 0.163 |  |
| **Recreation and tourism** | **Subtropical** | **best** | **0.74** | **0.54** | **0.88** | **0.023** | ***** |
| Societal awareness and values | Subtropical | best | 0.59 | 0.37 | 0.77 | 0.420 |  |
| **Socio-economic development** | **Subtropical** | **best** | **0.82** | **0.64** | **0.92** | **0.002** | ****** |
| Technology and innovation | Subtropical | best | 0.43 | 0.24 | 0.64 | 0.507 |  |
| **Trade and transport** | **Subtropical** | **best** | **0.93** | **0.81** | **0.98** | **0.000** | ******* |
|  |  |  |  |  |  |  |  |
| Biodiversity loss and degradation | Temperate | best | 0.62 | 0.41 | 0.80 | 0.271 |  |
| **Climate change** | **Temperate** | **best** | **0.80** | **0.61** | **0.91** | **0.004** | ****** |
| Communication and outreach | Temperate | best | 0.40 | 0.21 | 0.61 | 0.338 |  |
| Cooperation, legislation and agreements | Temperate | best | 0.59 | 0.37 | 0.77 | 0.420 |  |
| **Eutrophication and pollution** | **Temperate** | **best** | **0.71** | **0.50** | **0.86** | **0.048** | ***** |
| Human population size and migration | Temperate | best | 0.68 | 0.47 | 0.84 | 0.092 |  |
| IAS management | Temperate | best | 0.33 | 0.17 | 0.55 | 0.122 |  |
| IAS science | Temperate | best | 0.40 | 0.21 | 0.61 | 0.338 |  |
| Land-use and land use-change | Temperate | best | 0.65 | 0.44 | 0.82 | 0.163 |  |
| Ocean acidification | Temperate | best | 0.49 | 0.29 | 0.70 | 0.941 |  |
| **Recreation and tourism** | **Temperate** | **best** | **0.71** | **0.50** | **0.86** | **0.048** | ***** |
| Societal awareness and values | Temperate | best | 0.46 | 0.26 | 0.67 | 0.712 |  |
| **Socio-economic development** | **Temperate** | **best** | **0.77** | **0.57** | **0.89** | **0.010** | ***** |
| Technology and innovation | Temperate | best | 0.49 | 0.29 | 0.70 | 0.941 |  |
| **Trade and transport** | **Temperate** | **best** | **0.96** | **0.87** | **0.99** | **0.000** | ******* |
|  |  |  |  |  |  |  |  |
| Biodiversity loss and degradation | Tropical | best | 0.68 | 0.47 | 0.84 | 0.091 |  |
| **Climate change** | **Tropical** | **best** | **0.77** | **0.57** | **0.89** | **0.010** | ***** |
| Communication and outreach | Tropical | best | 0.62 | 0.41 | 0.80 | 0.271 |  |
| Cooperation, legislation and agreements | Tropical | best | 0.62 | 0.41 | 0.80 | 0.271 |  |
| **Eutrophication and pollution** | **Tropical** | **best** | **0.71** | **0.50** | **0.86** | **0.048** | ***** |
| **Human population size and migration** | **Tropical** | **best** | **0.82** | **0.64** | **0.92** | **0.002** | ****** |
| IAS management | Tropical | best | 0.56 | 0.35 | 0.75 | 0.608 |  |
| IAS science | Tropical | best | 0.62 | 0.41 | 0.80 | 0.271 |  |
| **Land-use and land use-change** | **Tropical** | **best** | **0.74** | **0.54** | **0.88** | **0.023** | ***** |
| **Ocean acidification** | **Tropical** | **best** | **0.71** | **0.50** | **0.86** | **0.048** | ***** |
| **Recreation and tourism** | **Tropical** | **best** | **0.77** | **0.57** | **0.89** | **0.010** | ***** |
| Societal awareness and values | Tropical | best | 0.62 | 0.41 | 0.80 | 0.271 |  |
| **Socio-economic development** | **Tropical** | **best** | **0.82** | **0.64** | **0.92** | **0.002** | ****** |
| Technology and innovation | Tropical | best | 0.56 | 0.35 | 0.75 | 0.608 |  |
| **Trade and transport** | **Tropical** | **best** | **0.93** | **0.81** | **0.98** | **0.000** | ******* |
|  |  |  |  |  |  |  |  |
| Biodiversity loss and degradation | Invertebrates | best | 0.62 | 0.41 | 0.80 | 0.268 |  |
| **Climate change** | **Invertebrates** | **best** | **0.85** | **0.68** | **0.94** | **0.001** | ******* |
| Communication and outreach | Invertebrates | best | 0.59 | 0.38 | 0.77 | 0.418 |  |
| **Cooperation, legislation and agreements** | **Invertebrates** | **best** | **0.71** | **0.51** | **0.86** | **0.045** | ***** |
| Eutrophication and pollution | Invertebrates | best | 0.68 | 0.47 | 0.84 | 0.088 |  |
| **Human population size and migration** | **Invertebrates** | **best** | **0.80** | **0.61** | **0.91** | **0.004** | ****** |
| IAS management | Invertebrates | best | 0.65 | 0.44 | 0.82 | 0.159 |  |
| IAS science | Invertebrates | best | 0.59 | 0.38 | 0.77 | 0.418 |  |
| **Land-use and land use-change** | **Invertebrates** | **best** | **0.71** | **0.51** | **0.86** | **0.045** | ***** |
| Ocean acidification | Invertebrates | best | 0.56 | 0.35 | 0.75 | 0.611 |  |
| **Recreation and tourism** | **Invertebrates** | **best** | **0.71** | **0.51** | **0.86** | **0.045** | ***** |
| Societal awareness and values | Invertebrates | best | 0.65 | 0.44 | 0.82 | 0.159 |  |
| **Socio-economic development** | **Invertebrates** | **best** | **0.77** | **0.57** | **0.89** | **0.009** | ****** |
| Technology and innovation | Invertebrates | best | 0.52 | 0.32 | 0.72 | 0.835 |  |
| **Trade and transport** | **Invertebrates** | **best** | **0.93** | **0.81** | **0.98** | **0.000** | ******* |
|  |  |  |  |  |  |  |  |
| Biodiversity loss and degradation | Microorganisms | best | 0.56 | 0.35 | 0.75 | 0.611 |  |
| **Climate change** | **Microorganisms** | **best** | **0.85** | **0.68** | **0.94** | **0.001** | ******* |
| Communication and outreach | Microorganisms | best | 0.59 | 0.38 | 0.77 | 0.418 |  |
| Cooperation, legislation and agreements | Microorganisms | best | 0.68 | 0.47 | 0.84 | 0.088 |  |
| **Eutrophication and pollution** | **Microorganisms** | **best** | **0.77** | **0.57** | **0.89** | **0.009** | ****** |
| **Human population size and migration** | **Microorganisms** | **best** | **0.74** | **0.54** | **0.88** | **0.021** | ***** |
| IAS management | Microorganisms | best | 0.59 | 0.38 | 0.77 | 0.418 |  |
| IAS science | Microorganisms | best | 0.59 | 0.38 | 0.77 | 0.418 |  |
| Land-use and land use-change | Microorganisms | best | 0.52 | 0.32 | 0.72 | 0.835 |  |
| Ocean acidification | Microorganisms | best | 0.52 | 0.32 | 0.72 | 0.835 |  |
| **Recreation and tourism** | **Microorganisms** | **best** | **0.85** | **0.68** | **0.94** | **0.001** | ******* |
| Societal awareness and values | Microorganisms | best | 0.59 | 0.38 | 0.77 | 0.418 |  |
| **Socio-economic development** | **Microorganisms** | **best** | **0.80** | **0.61** | **0.91** | **0.004** | ****** |
| Technology and innovation | Microorganisms | best | 0.52 | 0.32 | 0.72 | 0.835 |  |
| **Trade and transport** | **Microorganisms** | **best** | **0.98** | **0.90** | **1.00** | **0.000** | ******* |
|  |  |  |  |  |  |  |  |
| Biodiversity loss and degradation | Vertebrates | best | 0.68 | 0.47 | 0.84 | 0.088 |  |
| **Climate change** | **Vertebrates** | **best** | **0.74** | **0.54** | **0.88** | **0.021** | ***** |
| Communication and outreach | Vertebrates | best | 0.46 | 0.26 | 0.67 | 0.693 |  |
| Cooperation, legislation and agreements | Vertebrates | best | 0.49 | 0.29 | 0.69 | 0.926 |  |
| Eutrophication and pollution | Vertebrates | best | 0.42 | 0.24 | 0.64 | 0.487 |  |
| **Human population size and migration** | **Vertebrates** | **best** | **0.74** | **0.54** | **0.88** | **0.021** | ***** |
| IAS management | Vertebrates | best | 0.39 | 0.21 | 0.61 | 0.319 |  |
| IAS science | Vertebrates | best | 0.39 | 0.21 | 0.61 | 0.319 |  |
| Land-use and land use-change | Vertebrates | best | 0.59 | 0.38 | 0.77 | 0.418 |  |
| Ocean acidification | Vertebrates | best | 0.33 | 0.17 | 0.54 | 0.109 |  |
| Recreation and tourism | Vertebrates | best | 0.56 | 0.35 | 0.75 | 0.611 |  |
| Societal awareness and values | Vertebrates | best | 0.52 | 0.32 | 0.72 | 0.835 |  |
| **Socio-economic development** | **Vertebrates** | **best** | **0.85** | **0.68** | **0.94** | **0.001** | ******* |
| Technology and innovation | Vertebrates | best | 0.46 | 0.26 | 0.67 | 0.693 |  |
| **Trade and transport** | **Vertebrates** | **best** | **0.89** | **0.75** | **0.96** | **0.000** | ******* |
|  |  |  |  |  |  |  |  |
| Biodiversity loss and degradation | Vascular plants | best | 0.65 | 0.44 | 0.82 | 0.159 |  |
| **Climate change** | **Vascular plants** | **best** | **0.80** | **0.61** | **0.91** | **0.004** | ****** |
| Communication and outreach | Vascular plants | best | 0.68 | 0.47 | 0.84 | 0.088 |  |
| Cooperation, legislation and agreements | Vascular plants | best | 0.56 | 0.35 | 0.75 | 0.611 |  |
| **Eutrophication and pollution** | **Vascular plants** | **best** | **0.74** | **0.54** | **0.88** | **0.021** | ***** |
| **Human population size and migration** | **Vascular plants** | **best** | **0.77** | **0.57** | **0.89** | **0.009** | ****** |
| IAS management | Vascular plants | best | 0.52 | 0.32 | 0.72 | 0.835 |  |
| IAS science | Vascular plants | best | 0.46 | 0.26 | 0.67 | 0.693 |  |
| **Land-use and land use-change** | **Vascular plants** | **best** | **0.74** | **0.54** | **0.88** | **0.021** | ***** |
| Ocean acidification | Vascular plants | best | 0.33 | 0.17 | 0.54 | 0.110 |  |
| **Recreation and tourism** | **Vascular plants** | **best** | **0.74** | **0.54** | **0.88** | **0.021** | ***** |
| Societal awareness and values | Vascular plants | best | 0.68 | 0.47 | 0.84 | 0.088 |  |
| **Socio-economic development** | **Vascular plants** | **best** | **0.80** | **0.61** | **0.91** | **0.004** | ****** |
| Technology and innovation | Vascular plants | best | 0.46 | 0.26 | 0.67 | 0.693 |  |
| **Trade and transport** | **Vascular plants** | **best** | **0.93** | **0.81** | **0.98** | **0.000** | ******* |
|  |  |  |  |  |  |  |  |
| **Biodiversity loss and degradation** | **Freshwater** | **best** | **0.78** | **0.58** | **0.90** | **0.007** | ****** |
| **Climate change** | **Freshwater** | **best** | **0.87** | **0.72** | **0.95** | **0.000** | ******* |
| Communication and outreach | Freshwater | best | 0.57 | 0.36 | 0.76 | 0.526 |  |
| Cooperation, legislation and agreements | Freshwater | best | 0.69 | 0.48 | 0.85 | 0.071 |  |
| **Eutrophication and pollution** | **Freshwater** | **best** | **0.85** | **0.68** | **0.94** | **0.000** | ******* |
| **Human population size and migration** | **Freshwater** | **best** | **0.87** | **0.72** | **0.95** | **0.000** | ******* |
| IAS management | Freshwater | best | 0.47 | 0.27 | 0.68 | 0.799 |  |
| IAS science | Freshwater | best | 0.47 | 0.27 | 0.68 | 0.799 |  |
| **Land-use and land use-change** | **Freshwater** | **best** | **0.72** | **0.52** | **0.86** | **0.036** | ***** |
| **Recreation and tourism** | **Freshwater** | **best** | **0.83** | **0.65** | **0.93** | **0.001** | ****** |
| Societal awareness and values | Freshwater | best | 0.57 | 0.36 | 0.76 | 0.526 |  |
| **Socio-economic development** | **Freshwater** | **best** | **0.87** | **0.72** | **0.95** | **0.000** | ******* |
| Technology and innovation | Freshwater | best | 0.54 | 0.33 | 0.74 | 0.735 |  |
| **Trade and transport** | **Freshwater** | **best** | **0.91** | **0.78** | **0.97** | **0.000** | ******* |
|  |  |  |  |  |  |  |  |
| Biodiversity loss and degradation | Marine | best | 0.63 | 0.42 | 0.81 | 0.222 |  |
| **Climate change** | **Marine** | **best** | **0.85** | **0.68** | **0.94** | **0.000** | ******* |
| Communication and outreach | Marine | best | 0.41 | 0.22 | 0.62 | 0.394 |  |
| Cooperation, legislation and agreements | Marine | best | 0.54 | 0.33 | 0.74 | 0.735 |  |
| Eutrophication and pollution | Marine | best | 0.60 | 0.39 | 0.78 | 0.353 |  |
| **Human population size and migration** | **Marine** | **best** | **0.75** | **0.55** | **0.88** | **0.017** | ***** |
| IAS management | Marine | best | 0.41 | 0.22 | 0.62 | 0.394 |  |
| IAS science | Marine | best | 0.50 | 0.30 | 0.71 | 0.966 |  |
| Land-use and land use-change | Marine | best | 0.54 | 0.33 | 0.74 | 0.735 |  |
| Ocean acidification | Marine | best | 0.60 | 0.39 | 0.78 | 0.353 |  |
| Recreation and tourism | Marine | best | 0.57 | 0.36 | 0.76 | 0.526 |  |
| Societal awareness and values | Marine | best | 0.41 | 0.22 | 0.62 | 0.394 |  |
| **Socio-economic development** | **Marine** | **best** | **0.75** | **0.55** | **0.88** | **0.017** | ***** |
| Technology and innovation | Marine | best | 0.50 | 0.30 | 0.71 | 0.966 |  |
| **Trade and transport** | **Marine** | **best** | **0.96** | **0.87** | **0.99** | **0.000** | ******* |
|  |  |  |  |  |  |  |  |
| Biodiversity loss and degradation | Terrestrial | best | 0.66 | 0.45 | 0.83 | 0.130 |  |
| **Climate change** | **Terrestrial** | **best** | **0.83** | **0.65** | **0.93** | **0.001** | ****** |
| Communication and outreach | Terrestrial | best | 0.50 | 0.30 | 0.71 | 0.966 |  |
| Cooperation, legislation and agreements | Terrestrial | best | 0.57 | 0.36 | 0.76 | 0.526 |  |
| **Eutrophication and pollution** | **Terrestrial** | **best** | **0.75** | **0.55** | **0.88** | **0.017** | ***** |
| **Human population size and migration** | **Terrestrial** | **best** | **0.83** | **0.65** | **0.93** | **0.001** | ****** |
| IAS management | Terrestrial | best | 0.41 | 0.22 | 0.62 | 0.394 |  |
| IAS science | Terrestrial | best | 0.41 | 0.22 | 0.62 | 0.394 |  |
| **Land-use and land use-change** | **Terrestrial** | **best** | **0.75** | **0.55** | **0.88** | **0.017** | ***** |
| Recreation and tourism | Terrestrial | best | 0.66 | 0.45 | 0.83 | 0.130 |  |
| Societal awareness and values | Terrestrial | best | 0.47 | 0.27 | 0.68 | 0.799 |  |
| **Socio-economic development** | **Terrestrial** | **best** | **0.83** | **0.65** | **0.93** | **0.001** | ****** |
| Technology and innovation | Terrestrial | best | 0.44 | 0.25 | 0.65 | 0.580 |  |
| **Trade and transport** | **Terrestrial** | **best** | **0.95** | **0.84** | **0.98** | **0.000** | ******* |
|  |  |  |  |  |  |  |  |
| Biodiversity loss and degradation | Developed countries | best | 0.57 | 0.36 | 0.76 | 0.508 |  |
| **Climate change** | **Developed countries** | **best** | **0.75** | **0.55** | **0.88** | **0.017** | ***** |
| Communication and outreach | Developed countries | best | 0.34 | 0.17 | 0.56 | 0.160 |  |
| Cooperation, legislation and agreements | Developed countries | best | 0.44 | 0.25 | 0.66 | 0.608 |  |
| Eutrophication and pollution | Developed countries | best | 0.67 | 0.45 | 0.83 | 0.125 |  |
| Human population size and migration | Developed countries | best | 0.57 | 0.36 | 0.76 | 0.508 |  |
| IAS management | Developed countries | best | 0.31 | 0.15 | 0.53 | 0.089 |  |
| IAS science | Developed countries | best | 0.38 | 0.20 | 0.60 | 0.269 |  |
| Land-use and land use-change | Developed countries | best | 0.67 | 0.45 | 0.83 | 0.125 |  |
| Ocean acidification | Developed countries | best | 0.57 | 0.36 | 0.76 | 0.508 |  |
| Recreation and tourism | Developed countries | best | 0.61 | 0.39 | 0.79 | 0.341 |  |
| Societal awareness and values | Developed countries | best | 0.41 | 0.22 | 0.63 | 0.419 |  |
| **Socio-economic development** | **Developed countries** | **best** | **0.73** | **0.52** | **0.87** | **0.035** | ***** |
| Technology and innovation | Developed countries | best | 0.44 | 0.25 | 0.66 | 0.608 |  |
| **Trade and transport** | **Developed countries** | **best** | **0.95** | **0.85** | **0.98** | **0.000** | ******* |
|  |  |  |  |  |  |  |  |
| Biodiversity loss and degradation | Developing countries | best | 0.70 | 0.48 | 0.85 | 0.068 |  |
| **Climate change** | **Developing countries** | **best** | **0.85** | **0.69** | **0.94** | **0.000** | ******* |
| Communication and outreach | Developing countries | best | 0.61 | 0.39 | 0.79 | 0.341 |  |
| Cooperation, legislation and agreements | Developing countries | best | 0.67 | 0.45 | 0.83 | 0.125 |  |
| **Eutrophication and pollution** | **Developing countries** | **best** | **0.78** | **0.58** | **0.90** | **0.007** | ****** |
| **Human population size and migration** | **Developing countries** | **best** | **0.83** | **0.65** | **0.93** | **0.001** | ****** |
| IAS management | Developing countries | best | 0.57 | 0.36 | 0.76 | 0.508 |  |
| IAS science | Developing countries | best | 0.64 | 0.42 | 0.81 | 0.214 |  |
| **Land-use and land use-change** | **Developing countries** | **best** | **0.78** | **0.58** | **0.90** | **0.007** | ****** |
| Ocean acidification | Developing countries | best | 0.67 | 0.45 | 0.83 | 0.125 |  |
| **Recreation and tourism** | **Developing countries** | **best** | **0.78** | **0.58** | **0.90** | **0.007** | ****** |
| Societal awareness and values | Developing countries | best | 0.57 | 0.36 | 0.76 | 0.508 |  |
| **Socio-economic development** | **Developing countries** | **best** | **0.92** | **0.78** | **0.97** | **0.000** | ******* |
| Technology and innovation | Developing countries | best | 0.54 | 0.33 | 0.74 | 0.712 |  |
| **Trade and transport** | **Developing countries** | **best** | **0.96** | **0.87** | **0.99** | **0.000** | ******* |
|  |  |  |  |  |  |  |  |
| **Biodiversity loss and degradation** | **Emerging countries** | **best** | **0.75** | **0.55** | **0.88** | **0.017** | ***** |
| **Climate change** | **Emerging countries** | **best** | **0.83** | **0.65** | **0.93** | **0.001** | ****** |
| Communication and outreach | Emerging countries | best | 0.54 | 0.33 | 0.74 | 0.712 |  |
| Cooperation, legislation and agreements | Emerging countries | best | 0.61 | 0.39 | 0.79 | 0.341 |  |
| **Eutrophication and pollution** | **Emerging countries** | **best** | **0.81** | **0.62** | **0.91** | **0.003** | ****** |
| **Human population size and migration** | **Emerging countries** | **best** | **0.83** | **0.65** | **0.93** | **0.001** | ****** |
| IAS management | Emerging countries | best | 0.54 | 0.33 | 0.74 | 0.712 |  |
| IAS science | Emerging countries | best | 0.57 | 0.36 | 0.76 | 0.508 |  |
| **Land-use and land use-change** | **Emerging countries** | **best** | **0.75** | **0.55** | **0.88** | **0.017** | ***** |
| Ocean acidification | Emerging countries | best | 0.64 | 0.42 | 0.81 | 0.214 |  |
| **Recreation and tourism** | **Emerging countries** | **best** | **0.85** | **0.69** | **0.94** | **0.000** | ******* |
| Societal awareness and values | Emerging countries | best | 0.61 | 0.39 | 0.79 | 0.341 |  |
| **Socio-economic development** | **Emerging countries** | **best** | **0.88** | **0.72** | **0.95** | **0.000** | ******* |
| Technology and innovation | Emerging countries | best | 0.61 | 0.39 | 0.79 | 0.341 |  |
| **Trade and transport** | **Emerging countries** | **best** | **0.96** | **0.87** | **0.99** | **0.000** | ******* |

1. Worst-case scenario

| **Driver** | **System** | **Scenario** | **estimate** | **conf.low** | **conf.high** | **p.value** |  |
| --- | --- | --- | --- | --- | --- | --- | --- |
|  |  |  |  |  |  |  |  |
| **Biodiversity loss and degradation** | **Polar** | **worst** | **0.96** | **0.87** | **0.99** | **0.000** | ******* |
| **Climate change** | **Polar** | **worst** | **0.98** | **0.90** | **1.00** | **0.000** | ******* |
| **Communication and outreach** | **Polar** | **worst** | **0.91** | **0.78** | **0.97** | **0.000** | ******* |
| **Cooperation, legislation and agreements** | **Polar** | **worst** | **0.95** | **0.84** | **0.98** | **0.000** | ******* |
| **Eutrophication and pollution** | **Polar** | **worst** | **0.89** | **0.75** | **0.96** | **0.000** | ******* |
| **Human population size and migration** | **Polar** | **worst** | **0.85** | **0.68** | **0.94** | **0.001** | ******* |
| **IAS management** | **Polar** | **worst** | **0.98** | **0.90** | **1.00** | **0.000** | ******* |
| **IAS science** | **Polar** | **worst** | **0.93** | **0.81** | **0.98** | **0.000** | ******* |
| **Land-use and land use-change** | **Polar** | **worst** | **0.95** | **0.84** | **0.98** | **0.000** | ******* |
| **Ocean acidification** | **Polar** | **worst** | **0.80** | **0.61** | **0.91** | **0.004** | ****** |
| **Recreation and tourism** | **Polar** | **worst** | **0.95** | **0.84** | **0.98** | **0.000** | ******* |
| **Societal awareness and values** | **Polar** | **worst** | **0.96** | **0.87** | **0.99** | **0.000** | ******* |
| **Socio-economic development** | **Polar** | **worst** | **0.93** | **0.81** | **0.98** | **0.000** | ******* |
| **Technology and innovation** | **Polar** | **worst** | **0.87** | **0.71** | **0.95** | **0.000** | ******* |
| **Trade and transport** | **Polar** | **worst** | **0.98** | **0.90** | **1.00** | **0.000** | ******* |
|  |  |  |  |  |  |  |  |
| **Biodiversity loss and degradation** | **Subtropical** | **worst** | **0.99** | **0.92** | **1.00** | **0.000** | ******* |
| **Climate change** | **Subtropical** | **worst** | **0.99** | **0.92** | **1.00** | **0.000** | ******* |
| **Communication and outreach** | **Subtropical** | **worst** | **0.95** | **0.84** | **0.98** | **0.000** | ******* |
| **Cooperation, legislation and agreements** | **Subtropical** | **worst** | **0.96** | **0.87** | **0.99** | **0.000** | ******* |
| **Eutrophication and pollution** | **Subtropical** | **worst** | **0.99** | **0.92** | **1.00** | **0.000** | ******* |
| **Human population size and migration** | **Subtropical** | **worst** | **0.95** | **0.84** | **0.98** | **0.000** | ******* |
| **IAS management** | **Subtropical** | **worst** | **0.99** | **0.92** | **1.00** | **0.000** | ******* |
| **IAS science** | **Subtropical** | **worst** | **0.96** | **0.87** | **0.99** | **0.000** | ******* |
| **Land-use and land use-change** | **Subtropical** | **worst** | **0.98** | **0.90** | **1.00** | **0.000** | ******* |
| **Ocean acidification** | **Subtropical** | **worst** | **0.95** | **0.84** | **0.98** | **0.000** | ******* |
| **Recreation and tourism** | **Subtropical** | **worst** | **0.96** | **0.87** | **0.99** | **0.000** | ******* |
| **Societal awareness and values** | **Subtropical** | **worst** | **0.98** | **0.90** | **1.00** | **0.000** | ******* |
| **Socio-economic development** | **Subtropical** | **worst** | **0.96** | **0.87** | **0.99** | **0.000** | ******* |
| **Technology and innovation** | **Subtropical** | **worst** | **0.87** | **0.71** | **0.95** | **0.000** | ******* |
| **Trade and transport** | **Subtropical** | **worst** | **0.99** | **0.92** | **1.00** | **0.000** | ******* |
|  |  |  |  |  |  |  |  |
| **Biodiversity loss and degradation** | **Temperate** | **worst** | **0.99** | **0.92** | **1.00** | **0.000** | ******* |
| **Climate change** | **Temperate** | **worst** | **0.98** | **0.90** | **1.00** | **0.000** | ******* |
| **Communication and outreach** | **Temperate** | **worst** | **0.95** | **0.84** | **0.98** | **0.000** | ******* |
| **Cooperation, legislation and agreements** | **Temperate** | **worst** | **0.98** | **0.90** | **1.00** | **0.000** | ******* |
| **Eutrophication and pollution** | **Temperate** | **worst** | **0.98** | **0.90** | **1.00** | **0.000** | ******* |
| **Human population size and migration** | **Temperate** | **worst** | **0.91** | **0.78** | **0.97** | **0.000** | ******* |
| **IAS management** | **Temperate** | **worst** | **0.99** | **0.92** | **1.00** | **0.000** | ******* |
| **IAS science** | **Temperate** | **worst** | **0.95** | **0.84** | **0.98** | **0.000** | ******* |
| **Land-use and land use-change** | **Temperate** | **worst** | **0.96** | **0.87** | **0.99** | **0.000** | ******* |
| **Ocean acidification** | **Temperate** | **worst** | **0.87** | **0.71** | **0.95** | **0.000** | ******* |
| **Recreation and tourism** | **Temperate** | **worst** | **0.95** | **0.84** | **0.98** | **0.000** | ******* |
| **Societal awareness and values** | **Temperate** | **worst** | **0.98** | **0.90** | **1.00** | **0.000** | ******* |
| **Socio-economic development** | **Temperate** | **worst** | **0.98** | **0.90** | **1.00** | **0.000** | ******* |
| **Technology and innovation** | **Temperate** | **worst** | **0.89** | **0.75** | **0.96** | **0.000** | ******* |
| **Trade and transport** | **Temperate** | **worst** | **0.99** | **0.92** | **1.00** | **0.000** | ******* |
|  |  |  |  |  |  |  |  |
| **Biodiversity loss and degradation** | **Tropical** | **worst** | **0.99** | **0.92** | **1.00** | **0.000** | ******* |
| **Climate change** | **Tropical** | **worst** | **0.96** | **0.87** | **0.99** | **0.000** | ******* |
| **Communication and outreach** | **Tropical** | **worst** | **0.93** | **0.81** | **0.98** | **0.000** | ******* |
| **Cooperation, legislation and agreements** | **Tropical** | **worst** | **0.96** | **0.87** | **0.99** | **0.000** | ******* |
| **Eutrophication and pollution** | **Tropical** | **worst** | **0.99** | **0.92** | **1.00** | **0.000** | ******* |
| **Human population size and migration** | **Tropical** | **worst** | **0.93** | **0.81** | **0.98** | **0.000** | ******* |
| **IAS management** | **Tropical** | **worst** | **0.99** | **0.92** | **1.00** | **0.000** | ******* |
| **IAS science** | **Tropical** | **worst** | **0.96** | **0.87** | **0.99** | **0.000** | ******* |
| **Land-use and land use-change** | **Tropical** | **worst** | **0.99** | **0.92** | **1.00** | **0.000** | ******* |
| **Ocean acidification** | **Tropical** | **worst** | **0.96** | **0.87** | **0.99** | **0.000** | ******* |
| **Recreation and tourism** | **Tropical** | **worst** | **0.95** | **0.84** | **0.98** | **0.000** | ******* |
| **Societal awareness and values** | **Tropical** | **worst** | **0.98** | **0.90** | **1.00** | **0.000** | ******* |
| **Socio-economic development** | **Tropical** | **worst** | **0.96** | **0.87** | **0.99** | **0.000** | ******* |
| **Technology and innovation** | **Tropical** | **worst** | **0.87** | **0.71** | **0.95** | **0.000** | ******* |
| **Trade and transport** | **Tropical** | **worst** | **0.99** | **0.92** | **1.00** | **0.000** | ******* |
|  |  |  |  |  |  |  |  |
| **Biodiversity loss and degradation** | **Invertebrates** | **worst** | **0.99** | **0.92** | **1.00** | **0.000** | ******* |
| **Climate change** | **Invertebrates** | **worst** | **0.99** | **0.92** | **1.00** | **0.000** | ******* |
| **Communication and outreach** | **Invertebrates** | **worst** | **0.95** | **0.84** | **0.98** | **0.000** | ******* |
| **Cooperation, legislation and agreements** | **Invertebrates** | **worst** | **0.96** | **0.87** | **0.99** | **0.000** | ******* |
| **Eutrophication and pollution** | **Invertebrates** | **worst** | **0.96** | **0.87** | **0.99** | **0.000** | ******* |
| **Human population size and migration** | **Invertebrates** | **worst** | **0.93** | **0.81** | **0.98** | **0.000** | ******* |
| **IAS management** | **Invertebrates** | **worst** | **0.99** | **0.92** | **1.00** | **0.000** | ******* |
| **IAS science** | **Invertebrates** | **worst** | **0.96** | **0.87** | **0.99** | **0.000** | ******* |
| **Land-use and land use-change** | **Invertebrates** | **worst** | **0.99** | **0.92** | **1.00** | **0.000** | ******* |
| **Ocean acidification** | **Invertebrates** | **worst** | **0.91** | **0.78** | **0.97** | **0.000** | ******* |
| **Recreation and tourism** | **Invertebrates** | **worst** | **0.95** | **0.84** | **0.98** | **0.000** | ******* |
| **Societal awareness and values** | **Invertebrates** | **worst** | **0.98** | **0.90** | **1.00** | **0.000** | ******* |
| **Socio-economic development** | **Invertebrates** | **worst** | **0.98** | **0.90** | **1.00** | **0.000** | ******* |
| **Technology and innovation** | **Invertebrates** | **worst** | **0.89** | **0.75** | **0.96** | **0.000** | ******* |
| **Trade and transport** | **Invertebrates** | **worst** | **0.99** | **0.92** | **1.00** | **0.000** | ******* |
|  |  |  |  |  |  |  |  |
| **Biodiversity loss and degradation** | **Microorganisms** | **worst** | **0.91** | **0.78** | **0.97** | **0.000** | ******* |
| **Climate change** | **Microorganisms** | **worst** | **0.99** | **0.92** | **1.00** | **0.000** | ******* |
| **Communication and outreach** | **Microorganisms** | **worst** | **0.93** | **0.81** | **0.98** | **0.000** | ******* |
| **Cooperation, legislation and agreements** | **Microorganisms** | **worst** | **0.98** | **0.90** | **1.00** | **0.000** | ******* |
| **Eutrophication and pollution** | **Microorganisms** | **worst** | **0.96** | **0.87** | **0.99** | **0.000** | ******* |
| **Human population size and migration** | **Microorganisms** | **worst** | **0.89** | **0.75** | **0.96** | **0.000** | ******* |
| **IAS management** | **Microorganisms** | **worst** | **0.99** | **0.92** | **1.00** | **0.000** | ******* |
| **IAS science** | **Microorganisms** | **worst** | **0.98** | **0.90** | **1.00** | **0.000** | ******* |
| **Land-use and land use-change** | **Microorganisms** | **worst** | **0.96** | **0.87** | **0.99** | **0.000** | ******* |
| **Ocean acidification** | **Microorganisms** | **worst** | **0.87** | **0.71** | **0.95** | **0.000** | ******* |
| **Recreation and tourism** | **Microorganisms** | **worst** | **0.93** | **0.81** | **0.98** | **0.000** | ******* |
| **Societal awareness and values** | **Microorganisms** | **worst** | **0.98** | **0.90** | **1.00** | **0.000** | ******* |
| **Socio-economic development** | **Microorganisms** | **worst** | **0.96** | **0.87** | **0.99** | **0.000** | ******* |
| **Technology and innovation** | **Microorganisms** | **worst** | **0.96** | **0.87** | **0.99** | **0.000** | ******* |
| **Trade and transport** | **Microorganisms** | **worst** | **0.99** | **0.92** | **1.00** | **0.000** | ******* |
|  |  |  |  |  |  |  |  |
| **Biodiversity loss and degradation** | **Vertebrates** | **worst** | **0.99** | **0.92** | **1.00** | **0.000** | ******* |
| **Climate change** | **Vertebrates** | **worst** | **0.93** | **0.81** | **0.98** | **0.000** | ******* |
| **Communication and outreach** | **Vertebrates** | **worst** | **0.95** | **0.84** | **0.98** | **0.000** | ******* |
| **Cooperation, legislation and agreements** | **Vertebrates** | **worst** | **0.98** | **0.90** | **1.00** | **0.000** | ******* |
| **Eutrophication and pollution** | **Vertebrates** | **worst** | **0.89** | **0.75** | **0.96** | **0.000** | ******* |
| **Human population size and migration** | **Vertebrates** | **worst** | **0.89** | **0.75** | **0.96** | **0.000** | ******* |
| **IAS management** | **Vertebrates** | **worst** | **0.99** | **0.92** | **1.00** | **0.000** | ******* |
| **IAS science** | **Vertebrates** | **worst** | **0.96** | **0.87** | **0.99** | **0.000** | ******* |
| **Land-use and land use-change** | **Vertebrates** | **worst** | **0.95** | **0.84** | **0.98** | **0.000** | ******* |
| **Ocean acidification** | **Vertebrates** | **worst** | **0.87** | **0.71** | **0.95** | **0.000** | ******* |
| **Recreation and tourism** | **Vertebrates** | **worst** | **0.91** | **0.78** | **0.97** | **0.000** | ******* |
| **Societal awareness and values** | **Vertebrates** | **worst** | **0.98** | **0.90** | **1.00** | **0.000** | ******* |
| **Socio-economic development** | **Vertebrates** | **worst** | **0.96** | **0.87** | **0.99** | **0.000** | ******* |
| **Technology and innovation** | **Vertebrates** | **worst** | **0.91** | **0.78** | **0.97** | **0.000** | ******* |
| **Trade and transport** | **Vertebrates** | **worst** | **0.99** | **0.92** | **1.00** | **0.000** | ******* |
|  |  |  |  |  |  |  |  |
| **Biodiversity loss and degradation** | **Vascular plants** | **worst** | **0.99** | **0.92** | **1.00** | **0.000** | ******* |
| **Climate change** | **Vascular plants** | **worst** | **0.98** | **0.90** | **1.00** | **0.000** | ******* |
| **Communication and outreach** | **Vascular plants** | **worst** | **0.93** | **0.81** | **0.98** | **0.000** | ******* |
| **Cooperation, legislation and agreements** | **Vascular plants** | **worst** | **0.98** | **0.90** | **1.00** | **0.000** | ******* |
| **Eutrophication and pollution** | **Vascular plants** | **worst** | **0.99** | **0.92** | **1.00** | **0.000** | ******* |
| **Human population size and migration** | **Vascular plants** | **worst** | **0.93** | **0.81** | **0.98** | **0.000** | ******* |
| **IAS management** | **Vascular plants** | **worst** | **0.99** | **0.92** | **1.00** | **0.000** | ******* |
| **IAS science** | **Vascular plants** | **worst** | **0.96** | **0.87** | **0.99** | **0.000** | ******* |
| **Land-use and land use-change** | **Vascular plants** | **worst** | **0.98** | **0.90** | **1.00** | **0.000** | ******* |
| **Ocean acidification** | **Vascular plants** | **worst** | **0.77** | **0.57** | **0.89** | **0.009** | ****** |
| **Recreation and tourism** | **Vascular plants** | **worst** | **0.93** | **0.81** | **0.98** | **0.000** | ******* |
| **Societal awareness and values** | **Vascular plants** | **worst** | **0.98** | **0.90** | **1.00** | **0.000** | ******* |
| **Socio-economic development** | **Vascular plants** | **worst** | **0.96** | **0.87** | **0.99** | **0.000** | ******* |
| **Technology and innovation** | **Vascular plants** | **worst** | **0.89** | **0.75** | **0.96** | **0.000** | ******* |
| **Trade and transport** | **Vascular plants** | **worst** | **0.99** | **0.92** | **1.00** | **0.000** | ******* |
|  |  |  |  |  |  |  |  |
| **Biodiversity loss and degradation** | **Freshwater** | **worst** | **0.99** | **0.92** | **1.00** | **0.000** | ******* |
| **Climate change** | **Freshwater** | **worst** | **0.98** | **0.90** | **0.99** | **0.000** | ******* |
| **Communication and outreach** | **Freshwater** | **worst** | **0.95** | **0.84** | **0.98** | **0.000** | ******* |
| **Cooperation, legislation and agreements** | **Freshwater** | **worst** | **0.96** | **0.87** | **0.99** | **0.000** | ******* |
| **Eutrophication and pollution** | **Freshwater** | **worst** | **0.99** | **0.92** | **1.00** | **0.000** | ******* |
| **Human population size and migration** | **Freshwater** | **worst** | **0.93** | **0.81** | **0.98** | **0.000** | ******* |
| **IAS management** | **Freshwater** | **worst** | **0.98** | **0.90** | **0.99** | **0.000** | ******* |
| **IAS science** | **Freshwater** | **worst** | **0.98** | **0.90** | **0.99** | **0.000** | ******* |
| **Land-use and land use-change** | **Freshwater** | **worst** | **0.95** | **0.84** | **0.98** | **0.000** | ******* |
| **Recreation and tourism** | **Freshwater** | **worst** | **0.96** | **0.87** | **0.99** | **0.000** | ******* |
| **Societal awareness and values** | **Freshwater** | **worst** | **0.98** | **0.90** | **0.99** | **0.000** | ******* |
| **Socio-economic development** | **Freshwater** | **worst** | **0.96** | **0.87** | **0.99** | **0.000** | ******* |
| **Technology and innovation** | **Freshwater** | **worst** | **0.89** | **0.75** | **0.96** | **0.000** | ******* |
| **Trade and transport** | **Freshwater** | **worst** | **0.98** | **0.90** | **0.99** | **0.000** | ******* |
|  |  |  |  |  |  |  |  |
| **Biodiversity loss and degradation** | **Marine** | **worst** | **0.96** | **0.87** | **0.99** | **0.000** | ******* |
| **Climate change** | **Marine** | **worst** | **0.96** | **0.87** | **0.99** | **0.000** | ******* |
| **Communication and outreach** | **Marine** | **worst** | **0.95** | **0.84** | **0.98** | **0.000** | ******* |
| **Cooperation, legislation and agreements** | **Marine** | **worst** | **0.96** | **0.87** | **0.99** | **0.000** | ******* |
| **Eutrophication and pollution** | **Marine** | **worst** | **0.99** | **0.92** | **1.00** | **0.000** | ******* |
| **Human population size and migration** | **Marine** | **worst** | **0.89** | **0.75** | **0.96** | **0.000** | ******* |
| **IAS management** | **Marine** | **worst** | **0.99** | **0.92** | **1.00** | **0.000** | ******* |
| **IAS science** | **Marine** | **worst** | **0.93** | **0.81** | **0.98** | **0.000** | ******* |
| **Land-use and land use-change** | **Marine** | **worst** | **0.87** | **0.72** | **0.95** | **0.000** | ******* |
| **Ocean acidification** | **Marine** | **worst** | **0.91** | **0.78** | **0.97** | **0.000** | ******* |
| **Recreation and tourism** | **Marine** | **worst** | **0.93** | **0.81** | **0.98** | **0.000** | ******* |
| **Societal awareness and values** | **Marine** | **worst** | **0.98** | **0.90** | **0.99** | **0.000** | ******* |
| **Socio-economic development** | **Marine** | **worst** | **0.95** | **0.84** | **0.98** | **0.000** | ******* |
| **Technology and innovation** | **Marine** | **worst** | **0.87** | **0.72** | **0.95** | **0.000** | ******* |
| **Trade and transport** | **Marine** | **worst** | **0.99** | **0.92** | **1.00** | **0.000** | ******* |
|  |  |  |  |  |  |  |  |
| **Biodiversity loss and degradation** | **Terrestrial** | **worst** | **0.99** | **0.92** | **1.00** | **0.000** | ******* |
| **Climate change** | **Terrestrial** | **worst** | **0.98** | **0.90** | **0.99** | **0.000** | ******* |
| **Communication and outreach** | **Terrestrial** | **worst** | **0.95** | **0.84** | **0.98** | **0.000** | ******* |
| **Cooperation, legislation and agreements** | **Terrestrial** | **worst** | **0.98** | **0.90** | **0.99** | **0.000** | ******* |
| **Eutrophication and pollution** | **Terrestrial** | **worst** | **0.96** | **0.87** | **0.99** | **0.000** | ******* |
| **Human population size and migration** | **Terrestrial** | **worst** | **0.95** | **0.84** | **0.98** | **0.000** | ******* |
| **IAS management** | **Terrestrial** | **worst** | **0.99** | **0.92** | **1.00** | **0.000** | ******* |
| **IAS science** | **Terrestrial** | **worst** | **0.98** | **0.90** | **0.99** | **0.000** | ******* |
| **Land-use and land use-change** | **Terrestrial** | **worst** | **0.99** | **0.92** | **1.00** | **0.000** | ******* |
| **Recreation and tourism** | **Terrestrial** | **worst** | **0.95** | **0.84** | **0.98** | **0.000** | ******* |
| **Societal awareness and values** | **Terrestrial** | **worst** | **0.98** | **0.90** | **0.99** | **0.000** | ******* |
| **Socio-economic development** | **Terrestrial** | **worst** | **0.96** | **0.87** | **0.99** | **0.000** | ******* |
| **Technology and innovation** | **Terrestrial** | **worst** | **0.89** | **0.75** | **0.96** | **0.000** | ******* |
| **Trade and transport** | **Terrestrial** | **worst** | **0.99** | **0.92** | **1.00** | **0.000** | ******* |
|  |  |  |  |  |  |  |  |
| **Biodiversity loss and degradation** | **Developed countries** | **worst** | **0.99** | **0.92** | **1.00** | **0.000** | ******* |
| **Climate change** | **Developed countries** | **worst** | **0.98** | **0.90** | **1.00** | **0.000** | ******* |
| **Communication and outreach** | **Developed countries** | **worst** | **0.93** | **0.82** | **0.98** | **0.000** | ******* |
| **Cooperation, legislation and agreements** | **Developed countries** | **worst** | **0.98** | **0.90** | **1.00** | **0.000** | ******* |
| **Eutrophication and pollution** | **Developed countries** | **worst** | **0.99** | **0.92** | **1.00** | **0.000** | ******* |
| **Human population size and migration** | **Developed countries** | **worst** | **0.90** | **0.75** | **0.96** | **0.000** | ******* |
| **IAS management** | **Developed countries** | **worst** | **0.98** | **0.90** | **1.00** | **0.000** | ******* |
| **IAS science** | **Developed countries** | **worst** | **0.95** | **0.85** | **0.98** | **0.000** | ******* |
| **Land-use and land use-change** | **Developed countries** | **worst** | **0.96** | **0.87** | **0.99** | **0.000** | ******* |
| **Ocean acidification** | **Developed countries** | **worst** | **0.95** | **0.85** | **0.98** | **0.000** | ******* |
| **Recreation and tourism** | **Developed countries** | **worst** | **0.92** | **0.78** | **0.97** | **0.000** | ******* |
| **Societal awareness and values** | **Developed countries** | **worst** | **0.98** | **0.90** | **1.00** | **0.000** | ******* |
| **Socio-economic development** | **Developed countries** | **worst** | **0.96** | **0.87** | **0.99** | **0.000** | ******* |
| **Technology and innovation** | **Developed countries** | **worst** | **0.90** | **0.75** | **0.96** | **0.000** | ******* |
| **Trade and transport** | **Developed countries** | **worst** | **0.99** | **0.92** | **1.00** | **0.000** | ******* |
|  |  |  |  |  |  |  |  |
| **Biodiversity loss and degradation** | **Developing countries** | **worst** | **0.99** | **0.92** | **1.00** | **0.000** | ******* |
| **Climate change** | **Developing countries** | **worst** | **0.98** | **0.90** | **1.00** | **0.000** | ******* |
| **Communication and outreach** | **Developing countries** | **worst** | **0.95** | **0.85** | **0.98** | **0.000** | ******* |
| **Cooperation, legislation and agreements** | **Developing countries** | **worst** | **0.98** | **0.90** | **1.00** | **0.000** | ******* |
| **Eutrophication and pollution** | **Developing countries** | **worst** | **0.99** | **0.92** | **1.00** | **0.000** | ******* |
| **Human population size and migration** | **Developing countries** | **worst** | **0.95** | **0.85** | **0.98** | **0.000** | ******* |
| **IAS management** | **Developing countries** | **worst** | **0.99** | **0.92** | **1.00** | **0.000** | ******* |
| **IAS science** | **Developing countries** | **worst** | **0.98** | **0.90** | **1.00** | **0.000** | ******* |
| **Land-use and land use-change** | **Developing countries** | **worst** | **0.99** | **0.92** | **1.00** | **0.000** | ******* |
| **Ocean acidification** | **Developing countries** | **worst** | **0.96** | **0.87** | **0.99** | **0.000** | ******* |
| **Recreation and tourism** | **Developing countries** | **worst** | **0.96** | **0.87** | **0.99** | **0.000** | ******* |
| **Societal awareness and values** | **Developing countries** | **worst** | **0.98** | **0.90** | **1.00** | **0.000** | ******* |
| **Socio-economic development** | **Developing countries** | **worst** | **0.98** | **0.90** | **1.00** | **0.000** | ******* |
| **Technology and innovation** | **Developing countries** | **worst** | **0.90** | **0.75** | **0.96** | **0.000** | ******* |
| **Trade and transport** | **Developing countries** | **worst** | **0.99** | **0.92** | **1.00** | **0.000** | ******* |
|  |  |  |  |  |  |  |  |
| **Biodiversity loss and degradation** | **Emerging countries** | **worst** | **0.99** | **0.92** | **1.00** | **0.000** | ******* |
| **Climate change** | **Emerging countries** | **worst** | **0.99** | **0.92** | **1.00** | **0.000** | ******* |
| **Communication and outreach** | **Emerging countries** | **worst** | **0.95** | **0.85** | **0.98** | **0.000** | ******* |
| **Cooperation, legislation and agreements** | **Emerging countries** | **worst** | **0.98** | **0.90** | **1.00** | **0.000** | ******* |
| **Eutrophication and pollution** | **Emerging countries** | **worst** | **0.99** | **0.92** | **1.00** | **0.000** | ******* |
| **Human population size and migration** | **Emerging countries** | **worst** | **0.93** | **0.82** | **0.98** | **0.000** | ******* |
| **IAS management** | **Emerging countries** | **worst** | **0.99** | **0.92** | **1.00** | **0.000** | ******* |
| **IAS science** | **Emerging countries** | **worst** | **0.96** | **0.87** | **0.99** | **0.000** | ******* |
| **Land-use and land use-change** | **Emerging countries** | **worst** | **0.99** | **0.92** | **1.00** | **0.000** | ******* |
| **Ocean acidification** | **Emerging countries** | **worst** | **0.95** | **0.85** | **0.98** | **0.000** | ******* |
| **Recreation and tourism** | **Emerging countries** | **worst** | **0.96** | **0.87** | **0.99** | **0.000** | ******* |
| **Societal awareness and values** | **Emerging countries** | **worst** | **0.98** | **0.90** | **1.00** | **0.000** | ******* |
| **Socio-economic development** | **Emerging countries** | **worst** | **0.98** | **0.90** | **1.00** | **0.000** | ******* |
| **Technology and innovation** | **Emerging countries** | **worst** | **0.90** | **0.75** | **0.96** | **0.000** | ******* |
| **Trade and transport** | **Emerging countries** | **worst** | **0.99** | **0.92** | **1.00** | **0.000** | ******* |
